# Addressing discretization-induced bias in demographic prediction

**DOI:** 10.1093/pnasnexus/pgaf027

**Published:** 2025-01-30

**Authors:** Evan Dong, Aaron Schein, Yixin Wang, Nikhil Garg

**Affiliations:** Department of Computer Science, Cornell University, Ithaca, NY 14853, USA; Department of Statistics and Data Science Institute, University of Chicago, Chicago, IL 60637, USA; Department of Statistics, University of Michigan, Ann Arbor, MI 48109, USA; School of Operations Research and Information Engineering, Cornell Tech, New York, NY 10044, USA

## Abstract

Racial and other demographic imputation is necessary for many applications, especially in auditing disparities and outreach targeting in political campaigns. The canonical approach is to construct continuous predictions—e.g. based on name and geography—and then to often *discretize* the predictions by selecting the most likely class (argmax), potentially with a minimum threshold (thresholding). We study how this practice produces *discretization bias*. For example, we show that argmax labeling, as used by a prominent commercial voter file vendor to impute race/ethnicity, results in a substantial under-count of Black voters, e.g. by 28.2% points in North Carolina. This bias can have substantial implications in downstream tasks that use such labels. We then introduce a *joint optimization* approach—and a tractable *data-driven threshold* heuristic—that can eliminate this bias, with negligible individual-level accuracy loss. Finally, we theoretically analyze discretization bias, show that calibrated continuous models are insufficient to eliminate it, and that an approach such as ours is necessary. Broadly, we warn researchers and practitioners against discretizing continuous demographic predictions without considering downstream consequences.

**Significance Statement**

Racial and ethnicity data are used to audit disparities and to reach voters in political campaigns. When individual-level data are unavailable, academics and practitioners use predictive models. We find that the methods by which continuous probabilistic scores are discretized into single labels introduces substantial bias in the fraction of individuals labeled as a minority and their geographic distribution, even if the predictive model is optimal. These errors can significantly affect downstream tasks, leading to biased audits or erroneous decisions. We introduce optimization-based methods that can be unbiased, with empirically negligible loss in accuracy. Our work emphasizes the (i) importance of considering both distributional and individual-level accuracy and (ii) quantitatively evaluating error in algorithmic demographic imputation in important applications.

## Introduction

Knowing demographic characteristics of individuals, particularly race and ethnicity, is necessary for many important applications, for example: auditing disparities such as in lending and policing (1–6), making more fair and accurate decisions in voterfile-based polling and turnout campaigns (7–11), and decision-making more broadly (10, 12–14).

However, individual-level demographic data are not always available, and so researchers and practitioners *impute* (i.e. *predict*) these characteristics using available individual-level data, including in all the above-cited examples. Crucially, both individual-level *and* aggregate distributional prediction errors matter for many downstream tasks: in auditing the effect of voter ID laws on voting, for example, classifying a white *individual* as Black affects disparity estimates, but so does counting *overall* fewer Black potential voters in a geographic area.

The standard approach is: for *N* individuals, the user observes features $x_{1},\ldots ,x_{N}$ (e.g. name, location) but not the desired labels $y_{1},\ldots ,y_{N}$ (e.g. race/ethnicity). The user has access to a trained predictive model q(y,x)≈Pr(y∣x), for example, Bayesian Improved Surname Geocoding (BISG) (15), its variants (16–20), or other machine learning-based approaches (14, 21–23). A downstream task then uses discrete *decisions*${\hat{y}}_{1},\ldots ,{\hat{y}}_{N}$ —for example, to send targeted voter outreach information to individuals based on their predicted demographics, or to audit whether minoritized individuals are treated disparately.

Inaccurate models are known to affect downstream tasks (24); for example, Baines and Courchane (25) and Zhang (6) observe that inaccurate models lead to an *over-*estimate of disparities that rely on such labels. Argyle and Barber (21) find that miscalibration in BISG is further correlated with neighborhood-level income, affecting downstream estimates of voter turnout by race and income. A large literature thus pursues accurate probability models q(y,x) (Chin et al. (26) surveys this literature; see also Greengard and Gelman (18)); Argyle and Barber (21) propose a random forest model that uses additional features to postprocess BISG outputs.

We, in contrast, ask: what is the right way for the user to produce individual discrete labels ${\hat{y}}_{i}$, given access to a model that produces continuous $q(y,x_{i})$? What are the downstream consequences of different types of errors caused by the choices? A seemingly obvious choice is to assign for each point the argmax of its predictive distribution—i.e.


$${\hat{y}}_{i}\leftarrow \arg\;\max_{y}q(y,x_{i})$$

for each *i*. Indeed, this approach is adopted by many users (7, 9, 27, 28), especially practitioners and model developers. While this decision rule seems unassailable for producing a single label, it may lead to what was recently dubbed *argmax bias* (29) in the algorithmic fairness literature, when the empirical distribution of decisions overrepresents the most likely label. Intuitively, if a group overall composes a small fraction of the population, even a Bayesian optimal predictive model may rarely assign plurality probability for that group, even for individuals of that group. More generally, as we show, discretization methods have different individual and aggregate error properties, and so the discretization method should depend on the downstream task.

Our empirical context centers on administrative voter records data, (known as *voterfile data*) in the United States, which is widely used across academia and industry (8, 9, 27, 30–33). Such data compile the information collected by state voter registries and includes demographic information of registered voters, such as race or gender. Some state registries collect and make available demographic information that is self-reported by voters; however, many do not. As a result, campaigns and academics frequently use imputed race/ethnicity in tasks such as survey targeting, get-out-the-vote messaging, and measuring disproportionate impacts of voter roll purges (11). We analyze imputed labels in a widely used commercial voter file from TargetSmart^a^—which essentially uses argmax—as well as the North Carolina voter registry directly.

We find that the algorithmically imputed race/ethnicity of registered voters is substantially skewed white: e.g. in North Carolina, the algorithmically labeled fraction of *African-American* voters in the voter file is 28.2% less than the fraction of registered voters who self-report as *African-American*. This bias is due to both continuous model miscalibration (16.3% points) and argmax bias (11.9% points). This pattern extends to every state—argmax discretization *undercounts voters of color in 48 out of 50 states* in comparison to the fraction implied by the continuous scores $q(y,x_{i})$. Other discretization rules have their own error patterns. The commonly used *threshold* rule (only classifying individuals for whom $q(y,x_{i})\geq t$, e.g. for threshold t=80%) (2, 5, 13, 34) exacerbates this bias, especially geographic skews, while leaving many points unclassified. *Randomizing* decisions (*i* is labeled as *y* with probability $q(y,x_{i})$) correctly matches the predictive distribution, but is relatively inaccurate on individual labels.

Next, we introduce *optimization-based* approaches to generate discrete labels, that construct an integer program that outputs a label for each data point *i*, while balancing multiple error objectives. We primarily consider two objectives: (i) individual data point-level *accuracy* (labeling each data point according to its true class) and (ii) overall population-level *distributional fidelity*: where the marginal class distribution of decisions ${\hat{y}}_{i}$ should be close to some desired distribution (e.g. the marginal distribution implied by the model *q*, or, when available, external data on the true marginal distribution). For example, if two data points each have a probability 50% for each of the two classes, it may deterministically assign them different labels so that the overall class balance of decisions is even. Empirically, we find that our approach can eliminate discretization-induced bias, *even without using any information beyond model predictions q(y,x)*, with a negligible loss in individual-level accuracy. We further develop an efficient heuristic that can achieve similar performance, that can be interpreted as a *data-driven threshold* approach, informed by the optimization solutions on a small subset of the data.

Finally, we theoretically characterize *decision-making* (discretization) bias, as distinct from *predictive modeling bias*: how does amplification of the most likely class depend on the properties of the discretization procedure and the continuous scores, respectively? In Theorem 1, we show that argmax bias can emerge even when the continuous predictive model is itself *unbiased* (calibrated) and is tightly connected to *predictive uncertainty*. In Theorem 2, we analyze *decision-making* approaches to reduce bias: how should discrete decisions ${\hat{y}}_{i}$ be made from a Bayes optimal model of continuous probabilities $q(y,x_{i})$? We show that a joint optimization approach such as ours is *necessary* for optimal discretization in the presence of a distributional objective and predictive uncertainty.

Putting things together, we caution against the use of existing discrete labels, as commonly distributed in commercial voter files, for sensitive applications where such bias would affect results. For prior results that rely on analyzing discretized demographics, we urge considering how undercounting minority groups or geographically skewing their distribution would affect the results. We further answer the question: what should a researcher or practitioner do to prevent discretization-induced bias?

1. When possible, use the *continuous* scores instead of single discrete labels, as in (2, 3, 12, 35–38). For example, some auditing tasks can be conducted with sufficiently calibrated scores. In SI Appendix C.1, we show how using continuous scores recovers self-identified results in downstream analyses of voter turnout by race and income, while discretizing leads to substantial bias. However, for some tasks, such as individual-level outreach decisions, individual-level discrete decisions might either be required or be easier to integrate into practitioner analysis pipelines.
2. Improve the continuous model *q* when possible—e.g. by acquiring more data or changing the modeling approach. This is the predominant approach pursued in the demographic prediction literature (26). However, this approach is insufficient. As we make precise in Theorem 1, improving continuous predictive model accuracy reduces the bias; however, even unbiased Bayes optimal predictors can induce argmax bias, unless predictions have zero error (can *perfectly* identify the true label for each data point).
3. When discretizing, consider the downstream desiderata—e.g. is accuracy among the labeled set most important, or label representativeness?—and evaluate how label error may affect results. For example, Fraga (7, 9) count voters by imputed race and account for variance induced by the imputation process; they further discuss that bias as we measure here would lead to conservative estimates of differences in voter turnout induced by changes such as redistricting, though do not empirically quantify the potential effect on the results.
4. Finally, the optimization-based approaches developed here can flexibly discretize labels that have the error properties that best match the downstream desiderata. For example, we illustrate approaches that maximize accuracy while matching group distributions both overall and per geographic subarea. In our analysis in SI Appendix C.1 in estimating voter turnout, our optimization-based methods reduce bias compared to argmax discretization, without using any additional information.

### Methods and data summary

We briefly summarize our primary methods and data, with additional details in the model and methods sections. Consider a trained model (such as BISG) that outputs a vector of label probabilities $q(x_{i})$ for each data point $x_{i}$. A (potentially randomized) *decision (discretization) rule*$D\;:\;{△}_{Y}^{N}\to Y^{N}$ is a rule that, given a set of probability vectors $\{q(x_{i}){\}}_{i=1}^{N}$ associated with *N* data points, assigns each data point *i* a label ${\hat{y}}_{i}\in Y$.

#### Decision desiderata

There are two high-level desiderata: (i) decision accuracy, i.e. making the correct decision *for each data point*; (ii) ensuring that the *distribution* of assigned labels $\left\{{\hat{y}}_{i}\right\}$ matches some desired distribution, a notion that we will capture both via (a) the bias for each class *y* and (b) full distributional fidelity. For example, we may want to assign a correct demographic label for each potential voter (for more effective personalized get-out-the-vote campaigns) but also have the overall label distribution match the demographic makeup of the state so that we’re not under-counting minoritized groups when conducting overall analyses.

(i) To measure of decision accuracy, we use (one minus the) 0–1 loss, i.e. given true labels $y_{i}$ and decisions ${\hat{y}}_{i}$: $\text{acc}(y_{1\;:\;N},{\hat{y}}_{1\;:\;N})=\frac{1}{N}\sum_{i}^{N}I[{\hat{y}}_{i}=y_{i}].$

(iia) To measure bias, we use the distance between the marginal distribution of labels and a *reference* distribution $p_{\mathrm{ref}}$. Let ${\hat{p}}_{\mathrm{marg}}(y)$ denote the fraction of datapoints with the label *y*:


$${\hat{p}}_{\mathrm{marg}}(y,{\hat{y}}_{1\;:\;N})=\frac{1}{N}\sum_{i=1}^{N}1[{\hat{y}}_{i}=y].$$

Then, bias is how much a class *y* is amplified by labels relative to the reference:


$$\text{bias}(y,{\hat{y}}_{1\;:\;N},p_{\mathrm{ref}})≜{\hat{p}}_{\mathrm{marg}}(y,{\hat{y}}_{1\;:\;N})-p_{\mathrm{ref}}(y).$$

(iib) As a summary statistic, we will further consider distributional fidelity, the negative sum of the absolute value of bias across classes (equivalently, the negative of the $\ell_{1}$ distance between the label and reference distributions).


$$\text{fid}(p_{\mathrm{ref}},{\hat{y}}_{1\;:\;N})≜-\sum_{y\in Y}\left|\text{bias}(y,{\hat{y}}_{1\;:\;N},p_{\mathrm{ref}})\right|.$$

##### Aggregate posterior reference distribution

What is an appropriate reference distribution $p_{\mathrm{ref}}$? Of course, if we knew the *true* distribution of labels (e.g. the true fraction of each group in the voter file), we could measure and optimize fidelity with respect to it. However, this information is not often known. In this article, we will thus primarily compare to the **aggregate posterior**: for a given set $\left\{x_{i}\right\}$, what would the decision distribution be if we made *continuous* decisions corresponding to the continuous classifier *q*,


$$p_{\mathrm{agg}}^{q}(y,\{x_{i}\})=\frac{1}{N}\sum_{i}q(y,x_{i}).$$

When *q* is Bayes optimal, this choice counts the “correct” number of decisions for each class if one was not forced to make discrete decisions, and approaches the true distribution Pr(y) as N→∞. This approach thus (i) isolates bias due to the discretization process as opposed to miscalibration in the continuous classifier *q*; (ii) can be optimized for and empirically measured, given predictions from *q*; i.e. this approach requires no more information than the argmax rule.

Notably, one can also calculate conditional aggregate posteriors—for example, the fraction of labels for each group *within each county*, if we observe such geographic information for each data point.

#### Overview of decision rules

Several rules are used in academia and in practice:

**Argmax** rule assigns, for each data point, the most likely^b^ class: $D_{\mathrm{argmax}}(\{q(x_{i}\})≜\{{\hat{y}}_{i}=\arg\;\max_{y}q(y,x_{i})\}$.

**Threshold at***t* rule assigns a label only if the argmax class has a probability of at least *t*, i.e. ${\hat{y}}_{i}=\arg\;\max_{y}q(y,x_{i})$ if $\max_{y}q(y,x_{i})\geq t$, otherwise *i* is left uncoded.

**Thompson sampling** for each data point *i*, *samples* a class *y* based on the probabilities $q(y,x_{i})$, i.e. ${\hat{y}}_{i}=y$ with probability $q(y,x_{i})$, and so in expectation will have labels matching the aggregate posterior.

These rules are all *independent*: the assigned label for a data point *i* depends on $q(x_{i})$ but not on other data points or their probability vectors $\{q(x_{j}){\}}_{\;j\neq i}$.

In contrast, our proposed approach also includes joint decision optimization rules, which depend on the entire set of data points, as a solution to discretization bias.

**Integer optimization** rules directly optimize given objectives. We consider the rule that corresponds to solving the following optimization problem, for a given γ∈[0,1], data points $\left\{x_{i}\right\}$, and reference $p_{\mathrm{ref}}$:

(1) $$D^{\gamma }(\{q(x_{i})\},p_{\mathrm{ref}})={\arg\;\max}_{\left\{{\hat{y}}_{i}\right\}}\gamma \left(\frac{1}{N}\sum_{i=1}^{N}q({\hat{y}}_{i},x_{i})\right)+(1-\gamma )\text{fid}(p_{\mathrm{ref}},{\hat{y}}_{1\;:\;N}).$$

The rule balances, parameterized by *γ*, row-level scores (accuracy as measured by *q*) and distributional fidelity.

**Aggregate posterior matching.** A special case of the joint optimization decision rules defined in Eq. 1 is as γ→0, leading to a rule that maximizes accuracy subject to the constraint that distributional fidelity be as high as possible. We call such rules *matching* solutions, e.g. *aggregate posterior matching*. For our results, we use aggregate posteriors calculated at multiple geographic levels: nationally, throughout a state, or for each county within the state.

**Data-driven threshold heuristic.** One downside of the optimization-based approaches is that they may be computationally expensive for large datasets (requiring an integer constrained variable for each data point). Thus, we develop and evaluate the following heuristic: solve the integer optimization for a tractable batch of data. Then, on that batch, train a machine learning model with features being the scores $q(y,x_{i})$ and the labels being the optimization outputs ${\hat{y}}_{i}$. Finally, apply this model to the rest of the dataset. Once trained, this approach is as simple as existing threshold or argmax approaches, and so we refer to it a *data-driven threshold heuristic* (with vector thresholds implicitly defined by the machine learning model). In this work, we train this approach to approximate the aggregate posterior matching solution and integer programs.

**Fig. 1.**
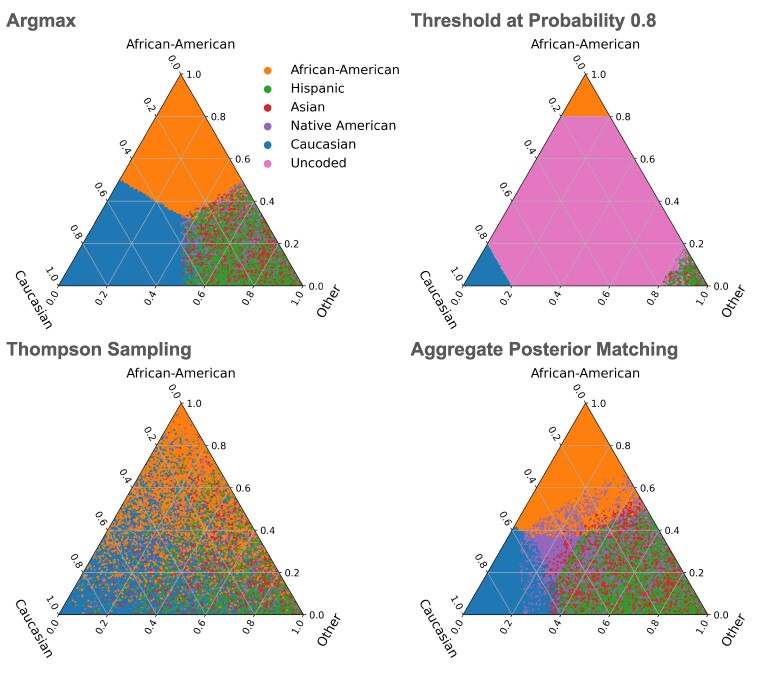
Comparison of different discretization methods. Each subfigure shows a 3D probability simplex, where individual points are colored according to the label assigned by the corresponding method. For example, in a), all the blue points (clustered in the bottom left) are assigned the *Caucasian* label, which has the highest class probability according to the continuous model *q* for that data point. We use a sample of points from the voter file, and *Hispanic*, *Asian*, and *Native American* probabilities are aggregated into *Other* (points primarily in the bottom right). Our approach in d) (posterior matching) matches the class distribution while maintaining individual data point level accuracy. a) Argmax, b) Threshold at Probability 0.8, c) Thompson Sampling, and d) Aggregate Posterior Matching.

Figure 1 illustrates labels as a function of the class probabilities for several decision rules. Intuitively, like Thompson sampling, aggregate posterior matching assigns a proportional number of data points to each class, which may require individual points to not be labeled by its most likely class. However, its labels are more in line with argmax labeling than is Thompson sampling.

#### Data and code

We evaluate a widely used commercial data file that provides both predicted continuous race/ethnic probabilities and a single discrete generated label for each individual. The voter file provides continuous probabilities for people along K=5 predicted race/ethnicity categories: *African-American*, *Asian*, *Caucasian*, *Hispanic*, and *Native American*.^c^

The labels also include an *Uncoded* category. Crucially, according to both the data dictionary and verified by us, the discrete labels are derived using the *argmax* rule, except for the *Uncoded* category.^d^ In our main text analysis, we relabel these *Uncoded* points via the argmax rule.^e^

We analyze the universe of available predictions and state voter files (N=261,547,234) across all 50 states and the District of Columbia. Furthermore, some states (such as North Carolina) additionally make available each voter’s *self-identified* race/ethnicity, as filled in on the voter registration form. Our data includes this information where available alongside the algorithmically generated predictions and discrete labels; in some of our analyses, we use self-report data as *ground truth labels*^f^ to measure true accuracy and to calculate a ground truth reference distribution $p_{\mathrm{ref}}(y)$. For these analyses in the main text, we filter our dataset of model predictions to include North Carolina only. This analysis contains N=6,374,636 individuals, after excluding the 2,312,356 individuals in NC without self-reported race and the 138,541 voters who self-report race as *Other*.^g^ In the SI Appendix, we replicate our primary analyses using fully public data and predictive modeling methods from Greengard and Gelman (18), as well as for other states in the commercial voter file. Our code is available at https://github.com/evan-dong/demographic-prediction-argmax-bias.

## Empirical results

###  

#### Discretization bias and voter of color representation

**Fig. 2.**
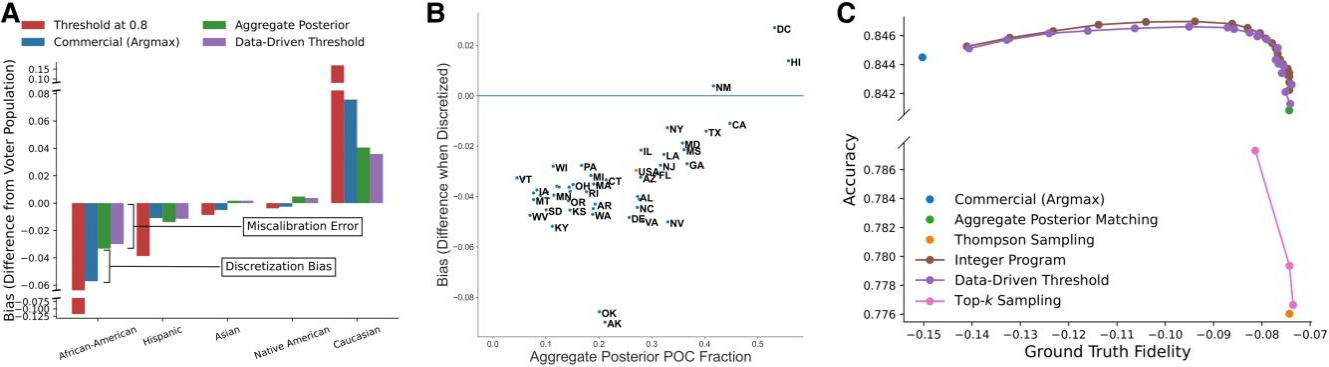
Bias (undercounting of voters of color) in the voter file. a) In North Carolina where ground truth self-reported data are available, the difference in counts for each group between each discretization method and the ground truth. The argmax method substantially undercounts Black voters in particular, with threshold methods further magnifying such bias. The bar marked “Aggregate Posterior” corresponds to the bias of both Thompson Sampling and Aggregate method; as these methods directly reflect proportions from the continuous model, its undercounting is due to the model’s miscalibration. The additional bias of argmax is thus the bias caused by discretizing the model scores. b) In all states plus Washington, DC, the discretization bias (difference between the fraction of voters of color in the discrete labels and the aggregation posterior fraction). Points below the horizontal line indicate a comparative underrepresentation of voters of color compared to the aggregate posterior—only in DC, Hawaii, and New Mexico does the discretization lead to an increase of the count of voters of color. Note that in DC and Hawaii, *Caucasian* is not the most common class: *African-American* (DC) and *Asian* (HI) are, respectively, and these classes are over-represented by argmax. New Mexico (NM) is the one exception where the argmax decision rule under-represents the most common class compared to the aggregate posterior. In b), some similarly clustered states are left unlabeled for visual clarity, and the overall effect in full voter file is marked in orange. In c), we plot the performance of different decision rules according to our two metrics. Notice that our optimization-based rules Pareto-dominate the sampling approach, and that the data-driven threshold approximates the full curve of integer programs quite closely. a) Differences in predicted and self-reported counts in NC, b) Per-state discretization bias, and c) Accuracy vs. Fidelity of rules in NC.

Argmax labeling substantially undercounts voters of color. Figure 2a shows—for North Carolina voters for whom self-identified labels are available—the bias (relative count) of each decision rule compared to this ground truth. *Every group except Caucasian* is under-counted by the argmax rule, and this under-counting is substantial. The predicted *African-American* population differs by over four hundred thousand out of a self-reported population count of 1,447,516; i.e. the discrete labels count over 28% fewer Black voters than self-reported figures. More precisely, compared to ground truth population distribution for each group, the labeled distribution has fewer *African-American* (−28.2%), *Asian* (−20.1%), *Hispanic* (−14.9%), and *Native American* (−32.8%) voters while inflating the *Caucasian* population by +10.1%. Furthermore, a large source of this undercounting is due to the *discretization* rule (argmax) in addition to miscalibration in the continuous classifier: the continuous classifier’s miscalibration causes 16.3% of the undercounting, with the remaining 11.9% caused by argmax discretization. For other (smaller) minority groups, the aggregate posterior (continuous classifier) *over-*counts the group, but the argmax bias is more severe. For example, for *Asian* voters, the calibration error *increases* counts by +10.8% points, but then argmax discretization undercounts, with additive bias of −30.9% points. For the smallest group (*Native American*), calibration error leads to overcounting of +64.4%, with additive argmax bias of −96.7% points relative to ground truth. Note that the errors partially cancel out for these groups, and the error sources are distinct: BISG-like methods may overcount these groups (perhaps due to geographic segregation and more distinct surnames), but the negative discretization bias is more severe as the groups become smaller. See SI Appendix C.2 for the full table of these errors by discretization method and classifier calibration curves using NC data.

These patterns extend to the other states. Since we do not have *self-identified ground truth* labels in every state, Fig. 2b compares the fraction of voters labeled as a person of color^h^ (any label except *Caucasian*) by the vendor, vs. the aggregate posterior fraction according to the same vendor’s continuous probabilities. In *nearly every state*, the fraction of people labeled as a person of color is *less than* the aggregate posterior—i.e. the argmax discretization rule comparatively undercounts people of color everywhere except New Mexico, Hawai’i, and DC. Notice the general trend in voter of color representation: the larger the white majority population, the larger the discretization bias.

### Mitigating bias with optimization-based decision rules

These biases can be mitigated via changing the discretization rule, with negligible loss in individual-level accuracy. Figure 2a shows that matching to the aggregate posterior (which does not require any more information than argmax) substantially reduces bias, and Fig. 2c shows accuracy vs. fidelity to the ground truth for each rule. Table 1 lists, for each decision rule, the accuracy with respect to the ground truth as well as distributional fidelity to both the ground truth distribution and the aggregate posterior.

**Table 1.** In North Carolina, the performance of different decision-making discretization rules.

	Accuracy with ground truth	Fidelity to ground truth	Fidelity to aggregate posterior
Threshold at 0.8 (67.9% not dropped)	**0.928**	− 0.352	− 0.293
Integer program, γ=0.9	0.846	− 0.079	− 0.019
Commercial (Argmax)	0.844	− 0.150	− 0.091
Aggregate posterior matching	0.841	** − 0.074**	** − 0.000**
Data-driven threshold matching heuristic	0.841	− 0.074	− 0.002
County-conditional aggregate posterior matching	0.834	− 0.074	− 0.000
Thompson sampling	0.776	− 0.074	− 0.000

Fidelity is compared both to the true marginal distribution (i.e. the self-reported voter population) and the aggregate posterior; differences between the two reflect probabilistic score miscalibration. The bolded values highlight the highest-performing method for each performance metric. Both sampling-based approaches are *strictly dominated* by joint decision-making rules, which achieve equal or better fidelity with substantially better accuracy. Furthermore, both matching approaches substantially improve on distributional fidelity (to either ground truth or aggregate posterior) than the argmax rule, with negligible loss in accuracy. Note that the aggregate posterior matching approach in particular uses no more data than the argmax approach. Metrics for the threshold rule are calculated only on labeled points, dropping 32.1% of the dataset.

Remarkably, our joint optimization-based rules have similar accuracy as the argmax rule (0.36% more 0–1 loss), with *substantially less* bias (from 15% to 7.4% with respect to ground truth). This is true for the matching rules (that exactly match the distribution implied by the continuous models), the data-driven threshold heuristic, and integer optimization rules that balance accuracy and distributional fidelity. Furthermore, the sampling-based approaches are highly suboptimal: Thompson sampling is substantially less accurate, with comparable distributional fidelity and bias to the joint rules.

Two aspects of the results are especially striking. (i) Our optimization-based decision rules outperform standard rules even though they are not given the true marginal distribution over classes, i.e. when it uses just the information given by the continuous classifier (and so can be implemented in every state, even when true population information is not available, just like the argmax rule). (ii) Even the extreme fidelity-prioritizing choice of γ→0 optimized by the matching approaches induces negligible accuracy loss, and so it seems appropriate for any downstream application (and notably, it is the opposite choice than the implicit γ=1 choice made by the current argmax approach).

Together, these results show that commercial voter files undercount voters of color, and this undercounting can be mitigated simply by changing how vendors convert continuous probabilities to discrete labels.

### Geographic skews

**Fig. 3.**
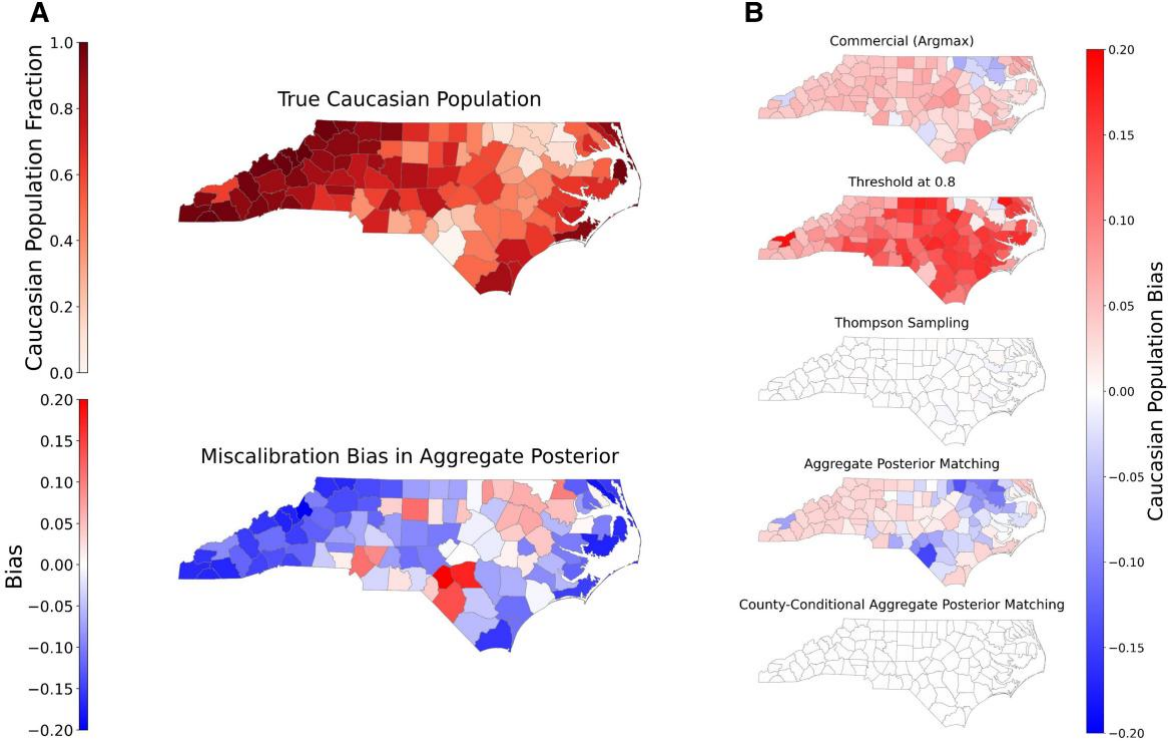
a) True *Caucasian* population in the voter file, and continuous model aggregate posterior bias compared to the true population. b) Per-county *Caucasian* bias in North Carolina, under different discretization methods—the underrepresentation of voters of color (i.e. bias overrepresenting the white population) when compared to the aggregate posterior. The most commonly used methods of thresholding and argmax further cause *geographic* skews—in many parts of the state, very few rows are classified as non-*Caucasian*, and the bias is largest in counties with an already skewed population. In contrast, Thompson sampling and County-conditional aggregate posterior matching have no bias when compared to the aggregate posterior. Note that geographical correlations in both miscalibration and discretization. a) Voter file population and model miscalibration and b) Bias compared to per-county aggregate posterior.

Finally, we consider sub-state bias. Figure 3 shows, for each county in North Carolina, the bias (in terms of white voters compared to the aggregate posterior) for each method, as well as the true fraction of white voters in the voter file.

Argmax and Threshold rules in particular exacerbate geographic differences: majority-white areas are made even *more* so through the imputation process. Intuitively, this occurs both because of conditional geographic miscalibration (models such as BISG use geography, and so a Black voter in a majority-white area is given a higher white probability than someone with the same name in a majority-Black area) and the discretization process (as discussed above, argmax and thresholding approaches amplify the majority class). Such geographic skews may be especially important in discrimination auditing settings, as they may lead to poor identification of groups in areas where they are especially in the minority and may face the most discrimination.

As with overall bias, such geography conditional bias can be mitigated with our optimization-based discretization rules, with minimal loss in accuracy. For example, an optimization approach that matches the aggregate posterior *for each county* can exactly match each county’s demographic distribution according to the model, with minimal loss in overall accuracy. (We note, however, that matching overall distributions without matching to each county can further polarize labels, where most nonwhite voters are drawn from counties where such groups live.) In SI Appendix C.1 in extending the analysis of Argyle and Barber (21), we illustrate that these geographic biases correlate with neighborhood-level income and skew voter turnout estimates, and that they can be effectively addressed either by directly accurate continuous scores or by discretizing using our matching methods.

Finally, Fig. 3b also illustrates one benefit of Thompson sampling over all other discretization approaches: due to the sampling process, it naturally balances per-sub-group distributions without explicit optimization. This unbiasedness property may especially be important in some auditing or polling settings, despite the individual-level inaccuracy.

### Result robustness and replication

The SI Appendix contains replications using public voterfile data and models from Greengard and Gelman (18). All results qualitatively replicate. Our replication code repository reproduces our results using this public dataset. Notably, one of their models (BISG on the voter file) is approximately calibrated for *White* and *Black*—i.e. the aggregate posterior is correct in terms of the fraction of *White* and *Black* voters. As we theoretically analyze below, we find that even this model leads to substantial discretization bias using standard approaches. In the appendix, we also show robustness of our results to alternative analysis choices, discretization methods, and subsets of the data.

## Theoretical characterization of argmax bias and joint decision-making

Motivated by the empirical results, we now characterize *decision-making* bias, as distinct from *predictive modeling bias*: how does amplification of the most likely class depend on the properties of the discretization procedure and the continuous scores, respectively?

In Theorem 1, we show that discretization bias can emerge even when the continuous predictive model is itself *unbiased* (calibrated), and is tightly connected to *predictive uncertainty*. As intuition, consider the no information regime: if the features provide no additional information over the overall class probability, then even for the Bayes optimal classifier we have $q(y,x_{i})=\mathrm{Pr}(y)$ for all *i*, and so all points get assigned label ${\hat{y}}_{i}=z$, the argmax class. On the other extreme of full information, Bayes optimal classifiers induce no argmax bias amplification, as they can identify the correct class for each data point. In the middle, we show that bias amplification is bounded by the mean average error of the continuous classifier.

In Theorem 2, we analyze *decision-making* approaches to reduce bias: how should discrete decisions ${\hat{y}}_{i}$ be made from a Bayes optimal model of continuous probabilities $q(y,x_{i})$? We show that independent decision-making is *necessarily* suboptimal in the presence of a distributional objective—any such policy, even those designed to meet distributional constraints (e.g. Thompson sampling), is Pareto dominated in terms of expected accuracy and distributional fidelity.

Proofs are in Appendix B.1.

### Formal definitions

There is a decision-making (classification) setting with discrete classes y∈Y, where |Y|=K≥2, with the *prior* class distribution Pr(y). There are *N* data points at the decision (test) time. For each data point *i*, we observe features $x_{i}\in X$, where the data and the unobserved true label $y_{i}$ are drawn from the joint distribution $(X,Y)∼F_{XY}$, where $F_{X},F_{Y}$ are both nonconstant. We distinguish between *continuous predictions*$q(y,x_{i})\in [0,1]$ and *discrete decisions*${\hat{y}}_{i}\in Y$. In our notation, we often omit distribution *F* and denote a set of data points as $x_{1\;:\;N}$.

#### Continuous classifier and discretization rules

Suppose we have a machine learning classifier *q* (e.g. trained on some other dataset) that makes continuous probability predictions for each class, given the data. Let q(y,x) denote the probability placed on class *y* given data *x*. For example, the Bayes optimal classifier is


$$q^{*}(y,x)≜\mathrm{Pr}(Y=y\;|\;X=x).$$

We will refer to *p* as the *prior* and *q* as our learned *posterior*. Let the simplex of possible probability vectors over the support Y be ${△}_{Y}$, and let $q(x)\in {△}_{Y}$ be the vector of probabilities $(q(y,x){)}_{y}$. Continuous classifiers are evaluated via their predictive performance. The expected **mean absolute error (MAE)** of the classifier *q* is:^i^


$$\text{MAE}(q)=E_{F_{XY}}\left[\frac{1}{N}\sum_{i}(1-q(y_{i},x_{i}))\right]=\int_{X}\sum_{y\in Y}\left(1-q(y,x)\right)\mathrm{Pr}(y|x)f(x)dx.$$

Decision (discretization) rules are as defined in Section 1.1. Our theoretical results distinguish between *independent* (e.g. argmax, thresholding, Thompson sampling) and *joint* (e.g. our optimization approach) rules. Formally, with independent rules, there exists a (potentially randomized) function *d* such that $D_{d}(\{q(x_{i})\})=\{{\hat{y}}_{i}=d(q(y,x_{i}))\}$.

#### Formal metrics and Pareto optimal decision-making

For our theoretical results, we consider the expectations of our accuracy, fidelity, and bias metrics, calculated over the joint distribution of the data $F_{XY}$ over datasets of size *N* (and any randomness in the decision rule). The *expected* accuracy of a decision rule *D* together with continuous predictor *q* is thus


(2) $${\text{ACC}}_{N}(D,q)=E_{F_{XY}}\left[\text{acc}(y_{1\;:\;N},D(\{q(x_{i})\}))\right].$$

Analogously, ${\text{BIAS}}_{N}(y^{*},D,q,p_{\mathrm{ref}})$ is the expectation of bias for a fixed chosen class *y*; that is,


(3) $${\text{BIAS}}_{N}(y,D,q,p_{\mathrm{ref}})=E_{F_{X}}\left[\text{bias}(y,D(\{q(x_{i})\}),p_{\mathrm{ref}})\right].$$

Accordingly, ${\text{FID}}_{N}(D,q,p_{\mathrm{ref}})$ is the analog to fid as BIAS is to bias; that is,


(4) $${\text{FID}}_{N}(D,q)=-\sum_{y\in Y}\left|{\text{BIAS}}_{N}(y,D,q,p_{\mathrm{ref}})\right|.$$

For a given data distribution *F*, continuous classifier *q*, reference distribution $p_{\mathrm{ref}}$, and dataset size, we want to make effective decisions. Given the multiple desiderata, we thus want Pareto optimal decision-making, i.e. to adopt a rule *D* that maximizes, for some γ∈(0,1],


(5) $$O_{N}^{\gamma }(D,q,p_{\mathrm{ref}})=\gamma {\text{ACC}}_{N}(D,q)+(1-\gamma ){\text{FID}}_{N}(D,q,p_{\mathrm{ref}}).$$

The weight *γ* is task-dependent, set by a practitioner’s expertise for whether accuracy or distributional fidelity is more important. When clear from context, we omit *q*, $p_{\mathrm{ref}}$, and *N* from the arguments for BIAS, $O^{\gamma }$, ACC, and FID.

### Theoretical results: argmax bias and continuous classifier

We first study the relationship between argmax bias and the properties of the continuous classifier. We show that discretization bias can occur even with unbiased (calibrated) continuous classifiers *q*, as long as it is uncertain (has nonzero error). Argmax bias depends on predictive uncertainty, i.e. how much information features *x* provide about the true class label.

The following result holds for all calibrated classifiers. A given classifier *q* is **calibrated** when its continuous predictions are correct on average, Pr(Y=y|q(y,x)=c)=c.^j^ The Bayes optimal classifier is calibrated.

Theorem 1. Consider calibrated classifier *q* and the argmax decision rule $D_{\mathrm{argmax}}$, with *N* datapoints and *K* classes, and N>K. Consider bias with respect to the aggregate posterior, $p_{\mathrm{ref}}=p_{\mathrm{agg}}$. Then, argmax bias is upper bounded by the predictive error of the classifier *q*:

(6) $$\text{BIAS}(y,D_{\mathrm{argmax}})\leq \text{MAE}(q).$$

The bound is tight: there exist $F_{XY}$ and *q* such that Eq. 6 holds with equality for the plurality class.

To prove this, we first construct an example in which bias equals MAE,^k^ and then show that any setting can be transformed into this example without violating the inequality.

Note that if the features provide no information about *Y*, the most common class is used for every label, maximizing bias. On the other extreme, if the classifier is perfect (MAE(q)=0), then each point is labeled according to its true class, and BIAS is also 0. While we primarily characterize positive bias (amplification of one class), Theorem 1 further implies lower bounds on negative bias, as bias across classes sums to 0. We elaborate on these corollaries in the SI Appendix.

**Fig. 4.**
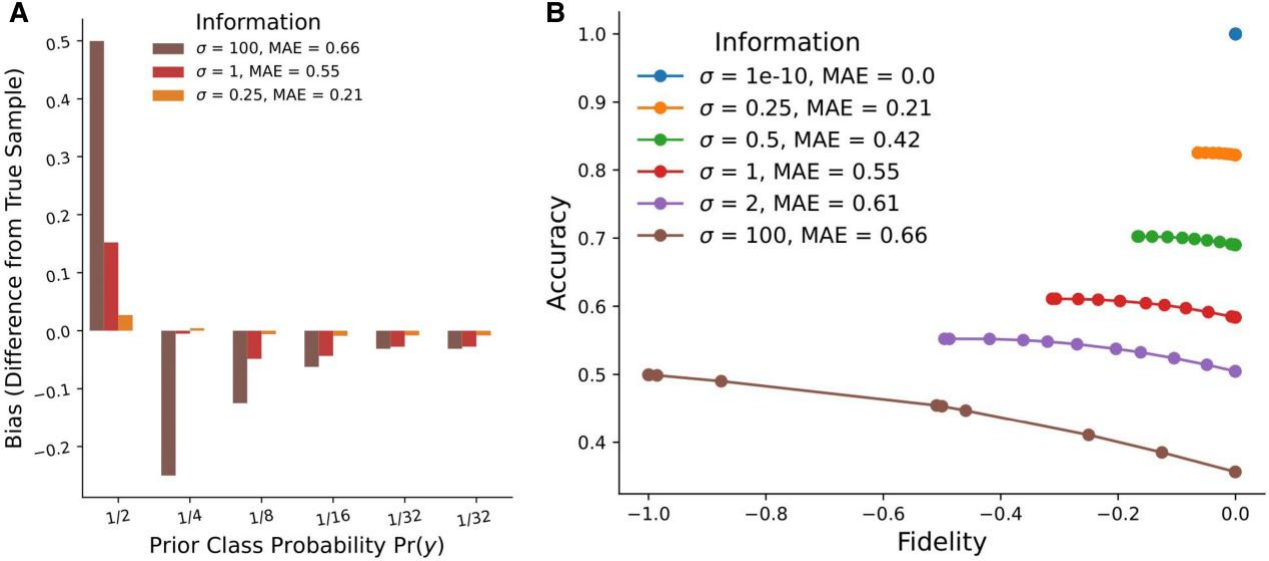
In simulation, discretized performance and argmax bias of the Bayes optimal classifier as a function of model accuracy, labeled by generating parameter *p* and MAE. The results reflect Theorem 1: as classifier accuracy increases (MAE decreases), argmax bias decreases, and both accuracy and distribution fidelity of optimal rules increase. Simulation details are described in Section 5. a) The argmax bias for each class and b) The Pareto curve of optimally performing decision rules.

We illustrate the main ideas of Theorem 1 on a simulated dataset: argmax bias (and more generally, the entire Pareto curve) depends on classifier accuracy, the MAE.^l^ Figure 4a shows $\text{BIAS}(y,D_{\mathrm{argmax}})$ for each class *y* (labeled with its class probability Pr(y)) as information changes. With little information, the argmax rule (completely) amplifies the highest probability class, and the bias decreases as classifier informativeness increases. Notice that in the lowest-accuracy setting, minority classes are missing from the output distribution entirely. Figure 4b shows the Pareto curves (with the argmax rule on one extreme of each Pareto curve). As information and therefore classifier accuracy increases, optimal decision-making improves both decision accuracy and distributional fidelity—as the uncertainty of *y* given *x*, as measured by the expected MAE, decreases, the Pareto curve shifts upwards.

This result clarifies the relationship between bias induced by the continuous model vs. the decision rule. Unlike the bias amplification observed in works such as Leino et al. (44), argmax bias occurs even for a calibrated classifier, one that places appropriate mass on each class, due to the threshold nature of argmax discrete decisions. We note that the observation that perfect accuracy eliminates bias amplification is in the work of Wang and Russakovsky (45), who further state that accuracy and bias are not always correlated; Theorem 1 shows that bias is upper bounded by calibrated classifier error and that the bound is tight. Overall, Theorem 1 suggests that improving model performance—calibrating its predictions, and improving accuracy—is aligned with mitigating argmax bias. However, it is insufficient: any less-than-perfect continuous model, even if unbiased by itself, can induce biased decisions. Empirically in the SI Appendix, we observe this in the voter file setting using the data and models of Greengard and Gelman (18); one model is approximately calibrated for *White* and *Black* people, but nevertheless discretization leads to undercounting of *Black* voters. Similarly, in extending the analysis of Argyle and Barber (21), we find that discretization biases turnout estimates even when using continuous model scores directly accurately reflect estimates using self-reported ground truth data.

### Results: individual discretization vs. joint optimization

Above, we study how the continuous classifier’s properties induce discretization bias, when using the argmax rule. We now study discrete decision-making: given an imperfect but Bayes optimal classifier, how can we make unbiased decisions? We show that joint decision-making, as in the program in Eq. 1, is necessary for Pareto optimality.

Define **nontrivial** reference distributions $P_{N}^{q}$ as follows: a reference distribution $p^{q}\;:\;\{x_{i}\}\to {△}_{Y}^{N}$ is in $P_{N}$ if it assigns mass at least $\frac{1}{N}$ to the most likely class in the dataset, i.e. at least 1 data point would be discretized to the most likely class $\arg\;\max_{y}\sum_{i}q(y,x_{i})$.^m^

Theorem 2. Consider Bayes optimal *q*, and N>K. For all joint distributions *F*:

- For every *γ*, $p_{\mathrm{ref}}$ there exists a joint decision-making rule that maximizes $O^{\gamma }(D,q,p_{\mathrm{ref}})$ in Eq. 5.
- The argmax decision rule $D_{\mathrm{argmax}}$ maximizes $O^{1}(D,q,p_{\mathrm{ref}})$, i.e. is accuracy maximizing.
- $D_{\mathrm{argmax}}$ is the *only* Pareto optimal independent decision rule for nontrivial $p_{\mathrm{ref}}\in P$. No independent decision rule *D* maximizes objective $O^{\gamma }$ for any *γ*, unless γ=1 and *D* agrees with $D_{\mathrm{argmax}}$ with probability 1.

Parts (i) and (ii) follow directly from the classifier being Bayes optimal—maximizing the objective for each given dataset can be formulated as an integer program, and the argmax rule maximizes expected accuracy for each data point. To prove part (iii), we show that any other independent rule, with positive probability, disagrees with argmax on every point in the dataset—and so we can improve both accuracy and fidelity by switching the decision for at least one point.

Figure 2c visualizes Theorem 2 on the voter file, showing the performance of the various decision rules that all use the same data and model *q*. Independent decision rules are substantially Pareto suboptimal compared to the optimal integer program—except the argmax rule, which optimizes accuracy at substantial cost of distributional fidelity. Note that in practice, without the Bayes optimal predictor, other decision rules may outperform and strictly dominate argmax, as seen in the peak of the integer program curve in Fig. 2c. Figure S1 further illustrates Theorem 2 on simulated data, in the SI Appendix.

We note that the joint decision-making approaches do not necessarily use *more information* than the argmax or other independent rules. For example, optimizing fidelity to the aggregate posterior simply requires the scores q(y,x)—the performance gain comes from jointly making decisions across points. On the other hand, we note that Theorem 2(ii) does not preclude other rules having higher accuracy than the argmax rule when the continuous model is not Bayes optimal, as shown in Fig. 2c. More generally, it is possible that, by incorporating domain knowledge such as the expected class distribution when available, that joint decision-rules can correct for model errors.^n^

## Discussion

Individual-level demographic imputation is a key algorithmic task across important applications. While much of the literature focuses on improving continuous predictive models, we show the importance of the next stage of discretizing continuous probabilities into individual labels. In particular, we show that the standard approaches of argmax and thresholding substantially skew the distribution of labels, especially as they might correlate with geography, decreasing the fraction of labeled minority groups. We further show that while increasing predictive modeling accuracy reduces (worst-case) bias, it is generally insufficient to eliminate such bias, even with perfectly calibrated models. We develop optimization-based discretization approaches that can match desired class distributions, with negligible loss in accuracy. More generally, (i) we caution against the use of standard discretization approaches, without considering the error properties that one’s downstream task requires; (ii) we emphasize discretization error as distinct from continuous model error: they have different causes, and may reinforce or partially cancel one another out in any given application.

While we empirically evaluate our optimization-based approach to balance individual-level accuracy and distributional fidelity, the approach is flexible to a range of objectives, such as precision, group specific false positives, and the count of individuals allocated to each class. Indeed, we emphasize that the choice of discretization method should be informed by the downstream task for which the labels will be used, and other considerations such as computational scalability and ethical considerations. For example, in some auditing tasks, it may be important to have high accuracy among the classified set (historically used to motivate thresholding approaches), along with geographic representation. In other settings, having the labels reflect the distribution even of *unknown* groups (e.g. that a proportional number of Black individuals who drive a specific type of car are classified as Black) may be more important than individual-level accuracy, suggesting the use of Thompson sampling. In some settings, it is possible to use continuous labels directly or to use self-reported demographic data—we recommend doing so when possible.

### Implications for the academic literature

We empirically find that discretization methods commonly used in the academic literature may undercount minority groups and skew their geographic distribution. First, research that *counts* voters (to e.g. measure the impact of laws or outreach effects) by imputed demographic group would relatively undercount minority groups. Second, even research that does not measure group size but rather measures effects *conditional* on an individual’s race/ethnicity would be affected by the biases we identify (e.g. “are [imputed] Black individuals more likely be poorly treated”). As Fig. 3b illustrates, the geographic distribution of predicted individuals does not reflect the ground truth distribution. Thus, instead of measuring an average statewide effect, such an analysis would instead relatively over-weight areas with larger minority populations and individuals with more demographically distinctive names. While such biases already occur with BISG-like continuous scoring, discretization (and especially the commonly used thresholding method) substantially exacerbates the bias, especially for small groups. An important avenue of future work is examining the extent to which these biases has affected prior findings. An immediate direction, for research that discretizes, is to analyze robustness of results by instead directly weighting by continuous model scores, with additional checks on how calibration errors would affect results.

As an example of how discretization affects downstream analyses, in SI Appendix C.1, we extend the analysis of Argyle and Barber (21), who demonstrate how misclassification bias of (discretized) BISG in the work of Imai and Khanna (47) correlates with socioeconomic status, affecting estimates of voter turnout by race and income. Argyle and Barber (21) propose to fix this bias by adjusting the BISG scores using a random forest model with additional individual and population-level features; their random forest model then implicitly discretizes its scores, using argmax. While Argyle and Barber (21) show that their discretized (more accurate) model substantially improves downstream analyses over discretized BISG, we show that directly using the continuous scores fully closes the gap between estimates and self-report data; our discretization methods also improve over the argmax discretized random forest.

### Implications for future work

Our work also has implications for future academic research on both using and developing demographic prediction models. As discussed above, research using demographic prediction should be specific on how errors (either continuous scoring or discretization) would affect results—whether by biasing counts or skewing the population that is studied. While some of these errors can be corrected (e.g. by adding geographic fixed effects), others may not be (e.g. skews due to surname distinctiveness). More generally, decomposing model scoring and discretization errors would clarify avenues for improvement. When using demographic prediction, both academically and in practice, we largely recommend using continuous scores directly, removing one source of error. When developing new prediction methods, researchers should separately report error metrics for continuous prediction (such as calibration curves and mean average error) and metrics (such as bias, 0–1 loss, and false positives/negatives) under the various discretization methods used in practice. We note that one practical barrier for practitioners may be that analytic pipelines are often easiest with a discrete single label per individual—we encourage package developers (e.g. those analyzing fairness metrics or conducting other analyses by group) to allow inputting probabilistic group scores; for use cases when the use of discrete labels is difficult to avoid, we encourage data provides (such as voter file providers) to discretize in a manner conducive to a diversity of downstream tasks, balancing individual and distributional accuracy.

### Small and intersectional groups

One limitation of our approach—indeed, of much of the demographic imputation literature—is the use of coarse racial/ethnic categories, and we advocate for methods that better deal with the full complexity of identity, while respecting privacy considerations (cf. Movva et al. (41)). This limitation is closely related to the question of how to proceed when the true group size of a subpopulation of interest (e.g. age, sex, and race subgroup) is unknown, as well as analogs in studying nonbinary gender. These questions are especially pertinent given the increased share of individuals in the United States identifying as multiracial (48). We foresee two challenges for research considering multirace, intersectional, or granular, or otherwise small group imputation: first, predictive models based on name and geography may be especially poor predictors; second, as our findings imply, the use of more, smaller categories would exacerbate argmax bias—conceptually and empirically, we find that negative discretization bias is relatively worse for smaller groups (Figs. 2b and S2b), as it is less likely that any individual’s argmax prediction is of that group. Such effects may occur even when the continuous model overcounts that group compared to self-identification, as in North Carolina for *Hispanic*, *Asian*, and *Native American* voters. In such cases, the discretization methods that we propose may reduce bias—especially if they have access to additional ground truth data—but may not be sufficient given the prediction challenge. The aggregate posterior implied by the continuous model (e.g. BISG) may also be especially biased, and our optimization-based solutions would reflect that bias. We anticipate that collecting and using self-identification data will be necessary for high-stakes analyses, as well as the development of hierarchical imputation (into both coarse and then granular categories) methods. Finally, we encourage researchers to account for increased uncertainty when analyzing small groups, as advocated by Himmelreich et al. (49) in the context of intersectionality in algorithmic fairness.

### Generalization of empirical results

Our empirical findings illustrate that overall bias results from an interaction of continuous model error and discretization bias—and so the empirical performance of a discretization method depends on the quality of the underlying predictive model. For example, in our replication using the data of Greengard and Gelman (18) in SI Appendix C.4, argmax (and thresholding) discretization continue to amplify the *White* group in all models. However, because two of the models’ continuous scores severely undercount the *White* group compared to self-identified ground truth, our aggregate posterior matching methods actually *worsen* ground truth distributional fidelity compared to argmax discretization. In yet other applications, self-identified “ground truth” data may also not be the gold standard, especially given the fluidity of racial identification (50). In such cases, our recommended approach is to decompose differences into its two components (difference tentative ground truth and model’s aggregate posterior), improve the continuous model when possible and necessary, and use an appropriate discretization method if necessary: no single discretization method generalizes for all possible use cases and underlying model performance.

### Discretization bias in other applications

While our empirical analysis focuses on demographic imputation in voter files, similar considerations appear in many other demographic imputation and algorithmic fairness tasks. For example, matching to the reference distribution could be interpreted as *group fairness* in algorithmic fairness settings, if each label *y* corresponds to a group, or *individual fairness* in recommendation settings, if each label corresponds to an individual producer or item. Note that in such applications, discretization may be necessary, as it corresponds to recommending an item to a user. Our optimization approach is thus related to the approaches of Seymen et al. (51) and Zhao et al. (52), and our work provides a characterization of such approaches, especially supporting joint optimization-based approaches over sampling in many applications. We discuss the connection to this literature further in SI Appendix A.

## Methodological details

**Argmax** The commercial dataset’s presupplied discrete labels did not appear to break ties in any totally consistent order. As a result, when labeling *Uncoded* data points (approximately 3.23% of the dataset) with argmax, we broke ties in the following order: *Caucasian*≻*African-American*≻*Hispanic*≻*Asian*≻*Native American*, which corresponds to both overall population size and is the least inconsistent with the provided labels. Tiebreaking has a negligible effect on results; there were a total of 390 such data points.

**Integer optimization** We solved integer programs using cvxpy (53), an open-source Python package, and Gurobi (54), used with a free academic license. In both simulation and empirical analyses, we calculated *γ* values between 0.8 and 0.99. Figure 2c uses increments of 0.01. We note that solving the optimization problem jointly over the full dataset may be computationally expensive; Seymen et al. (51) introduce a Lagrangian-based approach. In our empirical application, we solve this optimization problem in evenly sized *batches* of approximately 10,000. When using the aggregate posterior conditioned upon geographical location, these batches are grouped by county instead of uniform at random selection.

**Aggregate posterior matching** Matching to an exact distribution (i.e. the fidelity-maximizing one) becomes an assignment problem, solvable in polynomial time as a maximum weight matching problem on a bipartite graph, as in Abdollahpouri et al. (55). We use a bipartite max weight algorithm in Python to solve this problem, rounding the distribution to the closest integer number of labels to the fidelity-maximizing distribution.

**Data-driven threshold heuristic** We explored several different machine learning models to approximate aggregate posterior matching. Logistic regression, random forests, and support vector machines all achieve similar performance given one N≈10,000 training batch, with ≈99% of predicted labels being identical to those of the matching solution. Our reported results use a linear SVM, approximating both the aggregate posterior matching solution (Table 1 and Fig. 2c) and integer programs (Fig. 2c).

We further evaluate two rules in the Pareto curve diagram Fig. 2c and the SI Appendix.

**Top-*k* sampling** Top-*k* sampling is used in many modern applications, including large language models (56), and is equivalent to Thompson sampling between just the (renormalized) probabilities of the top *k* classes, with the remaining classes set to probability 0.

**True marginal population matching** In some settings, *true* aggregate population-level information may be available or well-estimated (e.g. through the census or surveys), or some other reference distribution may be chosen given normative motivations. We perform matching using the true makeup of self-reported race to demonstrate the effectiveness of our approach in utilizing additional information.

### Synthetic data generation

Our synthetic data setting for Figs. 4 and S1 is designed to illustrate our main ideas. We use K=6 classes (i.e. Y={1…6}), with the prior frequencies being sequential negative powers of 2, with the final two classes having equal frequency (i.e. {1/2,1/4,1/8,1/16,1/32,1/32}).

We simulate the features *x* as a *K*-dimensional vector; each dimension is drawn from a Normal distribution with variance $\sigma^{2}$, where the dimension corresponding to the true *y* has mean 1 and the remaining dimensions have mean 0. Thus, the variance $\sigma^{2}$ characterizes the informativeness of the data (lower *σ* means lower predictor MAE of the Bayes optimal classifier $q^{*}(y,x)=\mathrm{Pr}(y\;|\;x)$). This choice allows calculating the Bayes optimal classifier and to sample data. Data are generated by first sampling $y_{1\;:\;5,000}$ from the prior, and then sampling $x_{1\;:\;5,000}$ with the Pr(x|y) probabilities above. We draw N=5,000 points for each dataset. We calculate the Bayesian optimal posterior from the generating parameters and average results across 100 datasets.

## Supplementary Material

pgaf027_Supplementary_Data

## Data Availability

The replication dataset in SI Appendix C.1 is public at Barber and Argyle (57) and the result of work by Argyle and Barber (21). The replication dataset in SI Appendix C.4 is public at Greengard and Gelman (58) and the result of work by Greengard and Gelman (18). The specific code and jupyter notebook used for these analyses are available at https://github.com/evan-dong/demographic-prediction-argmax-bias. A more general repository of code with a jupyter notebook for other researchers and practitioners to discretize and analyze their own model outputs is at https://github.com/evan-dong/demographic-discretization. The commercial dataset used in our analysis is privately owned by TargetSmart, a political data and analytics company, a copy of which we accessed with a research license from PredictWise, a campaign analytics firm. We are unable to provide public access to this proprietary dataset. Researchers can apply for access to TargetSmart data by contacting TargetSmart at: https://targetsmart.com/contact-us/.

## Notes

a We received a research license to this data from PredictWise, a campaign analytics firm.

b Suppose ties are broken according to some consistent ordering over the classes. In the event of ties, we will use $\arg\;\max_{y}q(y,x)$ to denote the *single* class that is in the argmax set and is first in the consistent ordering among classes in the argmax set.

c These categories are imperfect and socially constructed, do not disambiguate race from ethnicity, and leave large granular variations uncaptured; to the extent that demographic-based analyses are important, we support the collection of more granular, self-reported data (39–43). We note that the voter file data treats *Hispanic* as mutually exclusive with racial categories, and furthermore that racial self-identification as *Native American* differs from legal tribal citizenship.

d The data dictionary states that an individual is marked as *Uncoded* when the model is not confident enough to make a prediction. From our analyses, it seems that the process of an individual being *Uncoded* cannot be simply described or reverse engineered as a threshold based rule; some uncoded rows have individual class probabilities of over 90%. We believe that validation from external data sources may play a role in an individual being *Uncoded*.

e We replicate all results in SI Appendix C.6 when we instead exclude these voters. As *Uncoded* data points are disproportionately predicted in probability to be voters of color, excluding these makes the population *less* diverse, *increasing* measured bias; results are qualitatively identical.

f Though we refer to these self-report labels as ground truth, we note that prior work has found that individuals may not be consistent in their reporting over time (7).

g Of course, excluding such individuals from consequential analyses could introduce further bias, speaking to the fraught nature of demographic imputation and coarse racial/ethnic data.

h While defining the boundaries of race and whiteness is always contentious, given the data at hand and these commercially used labels, our operational definition of voters of color includes *Hispanic* voters of any race.

i Note that, though related, mean average error of the continuous predictor q(y,x) is not equivalent to expected zero-one loss of the discrete decisions. The latter, our measure of decision accuracy, also depends on how the continuous predictor is discretized.

j For all *c* of nonzero measure: $\forall c\in \{c\;:\;|\int_{\left(x,y\right)}I[q(y,x)=c]f(x,y)d(y,x)|>0\}$. Further note that calibration implies Bayesian consistency, i.e. the continuous model cumulatively assigns the proper mass to each class *y*: $E_{X}[q(y,x)]=\mathrm{Pr}(y)$.

k For an argmax class *z*, we have $q(z,x)\in \{\frac{1}{K},1\}$ when $D_{\mathrm{argmax}}(q(x))=z$ and q(z,x)=0 otherwise.

l Our data simulation process is described in greater detail in Section 5; in short, we generate datasets with varying correlations between *X* and *Y* and calculate the Bayes optimal posterior $q^{*}$ in closed form.

m This technical condition eliminates the optimality of independent decision rules that trivially allocate every decision to some arbitrary fixed class, under a reference distribution that requires every point to be allocated to that class regardless of the continuous predictions *q*.

n For example, in Balachandar et al. (46), external information aids in estimating unbiased continuous predictive models when outcome data are missing.

## Preprints

This manuscript was posted on a preprint: https://doi.org/10.48550/arXiv.2405.16762.
