# Supplementary material for: Addressing discretization-induced bias in demographic prediction
Source: PNAS Nexus. 2025 Jan 30;4(2):pgaf027. doi: 10.1093/pnasnexus/pgaf027 (PMC11803427; doi:10.1093/pnasnexus/pgaf027)
Supplement: pgaf027_Supplementary_Data [file pgaf027_supplementary_data.pdf]

# Supplementary Material for "Addressing Discretization-Induced Bias in Demographic Prediction"

## A Extended Related Work

While we focus on demographic imputation as our primary application, the observation that algorithmic systems tend to *amplify* the most likely class has reappeared with different names across computational fields several times, with several proposed solutions. To this literature, we contribute an analysis of how different discretization approaches have different error properties, suggesting the use of optimization-based approaches tuned to one's specific desiderata.

### A.1 Argmax bias, calibration, and bias amplification across fields

Our work generally connects to work within algorithmic fairness and related fields. For example, if each class $y$ represents a (demographic) group, then distributional fidelity could represent a group fairness notion, with $p_{\text{ref}}(y) = \frac{1}{K}$ for all $y$, or corresponding to the class probabilities $p_{\text{ref}}(y) = \Pr(y)$.

Yee et al. [2021] term "argmax bias" and study whether it affects image cropping algorithms used by Twitter (prioritizing male faces in image previews). In image labeling tasks, Zhao et al. [2017] find that standard algorithms tend to over-label an agent as a *woman* when they are *cooking* in an image, with similar gender "bias amplification" occurring for other verbs such as *shopping* and *coaching*. For example, Zhao et al. [2017] find that when the fraction of training set images that are of women cooking Pr(women) is greater than that of men cooking, the fraction of times an image labeler labels a person who is cooking as a woman is even *greater*, $\Pr(\hat{y}_i = \text{women}) > \Pr(\text{women})$. Others then refine and expand on such bias [Wang and Russakovsky, 2021]. Similarly, Stanovsky et al. [2019] find that standard machine translation algorithms amplify gender stereotypes (e.g., assign a gender-neutral *doctor* as *man* when translating to a language with gendered nouns). In recommendation systems, Steck [2018] proposes the notion of "calibration," to counter the phenomenon that a user who prefers horror movies 70% of the time and comedy movies 30% of the time would only be recommended horror movies (this same notion of calibration is also present in algorithmic fairness settings). In biological engineering, deterministic binning of measured fluorescence systematically biases population estimates [Trippe et al., 2022]. The fundamental phenomenon across settings is that absent sufficient context-specific information for a specific data point, learning systems that optimize for accuracy will default to the globally most likely outcome. Many papers study related biases in the various contexts [Birhane et al., 2022, Costanza-Chock et al., 2022, Garg et al., 2018, 2021, Guo et al., 2021, Hall et al., 2022, Jacobs and Wallach, 2021, Jagadeesan et al., 2023a,b, Jia et al., 2020, Mansoury et al., 2020, Peng et al., 2024, Taori and Hashimoto, 2023, Zhao et al., 2023]; see also Arvind Narayanan's summary in a Twitter thread [Narayanan, 2021] on argmax bias.

Given this widespread identification of the problem (and more generally, a focus on the algorithmic fairness notion of *group fairness*), there has been a large literature studying its causes and proposing a range of solutions.

*The role of the continuous model $q$.* The predominant focus in algorithmic fairness, like in demographic imputation, has been on the continuous predictive model $q(y, x) \approx \Pr(Y|X)$ and its inputs [Black et al., 2023]. The predictive model itself can be biased, i.e., for the argmax class $z$, we have that $q(z, x) > \Pr(Y = z|X = x)$. Leino et al. [2019] show that bias amplification in the continuous modeling stage can be caused by inductive biases of stochastic gradient descent; they propose feature selection pre-processing as a solution. More generally, a large literature in fair machine learning aims to change continuous predictions $q(y, x)$

[Caton and Haas, 2020], such as by constrained optimization approaches during training (in-processing) or resampling training data (pre-processing). Chen et al. [2018] argue that "unfairness induced by inadequate samples sizes or unmeasured predictive variables should be addressed through data collection, rather than by constraining the model," and Cai et al. [2020] and Noriega-Campero et al. [2019] propose active information acquisition approaches to counter unfairness.

*The role of discrete decision-making.* Bias amplification can also occur in the discrete decision-making stage, as the term *argmax bias* [Yee et al., 2021] suggests – as discussed above, even if the predictive model is optimal, i.e., $q(y, x) = \Pr(Y|X)$, argmax discretization can amplify the most common class. Recognizing this cause, another set of technical approaches is to instead intervene at the *decision point*, when going from a continuous score $q(y|x)$ to a discrete label or decision $\hat{y}$.[1] We distinguish these technical solutions into two classes, *independent* and *joint*.

**Independent decision-making** The first approach class is to change the mapping from $q(y, x_i)$ to $\hat{y}_i$, *independently* for each data point $i$. By far the most commonly proposed technical fix in the algorithmic fairness community at the decision point is to *sample* decisions – for each data point $x$, probabilistically assign it a decision $y$ as a function of the continuous score $q(y, x)$. This approach can eliminate argmax bias – each class occurs in the correct proportions – but there may be substantial inaccuracy: a class with a posterior probability of only 10% would be the assigned decision for about 10% of the data points. Sampling is proposed prominently in the Twitter image cropping work that termed argmax bias [Yee et al., 2021], and is also proposed as a solution to such biases by Narayanan [2021], in natural language processing [Hendricks et al., 2018], biological engineering [Trippe et al., 2022], in admission settings with heterogeneous information [Liu and Garg, 2021], and algorithmic fairness generally [Caton and Haas, 2020]. Despite sampling's popularity, there is a sense it is inadequate. As Noriega-Campero et al. [2019] highlight when proposing an information acquisition approach in algorithmic fairness,

> "*Recent work has proposed optimal post-processing methods that randomize classification decisions for a fraction of individuals, to achieve fairness measures related to parity in errors and calibration. These methods, however, have raised concern due to the information inefficiency, intra-group unfairness, and Pareto sub-optimality*"

Another independent approach involves setting fixed thresholds and only discretizing a data point if the class probability is sufficiently high; if a data point does not meet any threshold, it is excluded [Adjaye-Gbewonyo et al., 2014, Chen et al., 2019, Consumer Financial Protection Bureau, 2014, Zhang, 2018]. This approach is often used for demographic imputation; Adjaye-Gbewonyo et al. [2014] pick different thresholds per group in a health care context.

**Joint decision-making** An alternative approach is to *jointly* make decisions across the entire dataset. Seymen et al. [2021] (for calibrated recommendations) and Zhao et al. [2017] (to reduce bias amplification in NLP) formulate similar integer optimization problems that jointly take scores $\{q(y, x_i)\}$ and output labels that balance maximizing individual-level accuracy and matching a class distribution. Abdollahpouri et al. [2023] formulate the extreme case of prioritizing matching the class distribution as an efficient max flow problem. As opposed to sampling approaches (and independent approaches more generally), joint decision-making is rarely even mentioned as a solution to argmax bias or in algorithmic fairness broadly –

[1] We note that there are also non-technical solutions. One especially relevant approach highlighted in the context of argmax bias is to delay algorithmic decisions or delegate to a human: if the problem is caused by discretizing continuous probabilities $\Pr(Y|X)$ into discrete decisions $\hat{y}$, then it can be avoided by not making discrete decisions. In the Twitter image cropping case, for example, a proposed solution was to allow users to manually crop images for previews [Yee et al., 2021]. As Narayanan [2021] summarizes in the twitter thread:

> "*I don't know any effective technical fixes for the argmax issue. But a really good approach for handling model uncertainty is to show the user the possible outputs and ask them to choose. But this goes against the mantra of frictionless design and so it's very rarely deployed.*"

When possible, we believe that such an approach is effective and appropriate, as emphasized by Yee et al. [2021]. However, we also believe that technical interventions at the point of decision-making under uncertainty are often necessary, and so should be improved.

for example, it does not appear in a table of six solutions by Yee et al. [2021], or as a solution approach in a survey of fair machine learning [Caton and Haas, 2020], though sampling and thresholding approaches are extensively detailed.

*Our contribution to this literature.* What does our work contribute to this extensive literature? (1) We analyze decision-making bias as distinct from and in relation to predictive modeling biases, requiring decision-making interventions: decision-making bias amplification occurs even with Bayes optimal, unbiased predictive modeling unless predictions are perfect ($\Pr(Y = y \mid X = x) = 1$ for the true class $y$, for each $x$). Thus, improving continuous predictions such as via active information acquisition is useful – improving accuracy reduces (worst case) bias when using the argmax decision rule – but it is generally insufficient. (2) Within the set of decision-making interventions, our work suggests a substantial departure from the algorithmic fairness status quo away from sampling based approach, and we suggest that optimization based approaches are relatively underused. More generally, we urge distinguishing between *predictive modeling* and *decision-making* biases in both auditing and bias mitigation, and urge more intentional choice of the discretization procedure: discretization is ultimately a highly context dependent decision, for which tradeoffs such as importance of accuracy, bias, computational scalability, other metrics, and non-quantitative concerns should be considered.

### A.2 Demographic imputation

Individual-level race and ethnicity data are used across applications, including public health, algorithmic fairness, political science, criminal justice, and economics – especially for disparity auditing [Chen et al., 2019, Chin et al., 2023, DeLuca and Curiel, 2022, Diamond et al., 2019, Garg et al., 2018, Ghosh et al., 2021, Grumbach and Sahn, 2020, Pierson et al., 2020]. However, collecting ground truth self-reported data may face practical or legal barriers [Consumer Financial Protection Bureau, 2014]. As a result, several methods have been developed to impute race/ethnicity using proxy variables, often name and geographic location [Chintalapati et al., 2023, Elliott et al., 2009, Fiscella and Fremont, 2006, Imai et al., 2022, Voicu, 2018]. For example, Bayesian Improved Surname Geocoding (BISG) [Elliott et al., 2009], a widely used method, was developed to estimate and assess racial disparities. Notably, the US Consumer Financial Protection Bureau uses BISG as a proxy to detect discriminatory lending practices, as creditors are generally prohibited from collecting race and ethnicity information [Andrus et al., 2021, Chen et al., 2019, Consumer Financial Protection Bureau, 2014]. By and large, these demographic prediction methods are probabilistic classifiers that first produce continuous predictions; then, these continuous scores are discretized, either through an argmax rule or approximately so (as we verify in our commercial voter file), with probability thresholds [Adjaye-Gbewonyo et al., 2014, Chen et al., 2019, Diamond et al., 2019].

Our empirical case study is in the context of voter registration data files, which form a backbone of public opinion and political science research for both academic and electoral ends; see Ghitza and Gelman [2020] for an overview. Since not every US state makes public individual-level self-reported race/ethnicity information, commercially sold datasets come with predicted demographic data for each registered voter, generated through proprietary models [Ansolabehere and Hersh, 2012]; these imputed demographic data are often provided both as continuous probabilities and discrete single labels [Ghitza and Gelman, 2020].

Many academic analyses (and from our experience, political campaign practitioners) use a voter file's discrete race and ethnicity labels [Ansolabehere and Hersh, 2011, Fraga, 2016a,b, Stauffer and Fraga, 2022]. Similarly, many academic papers beyond voter file analyses use discrete labels, instead of continuous probability scores [Adjaye-Gbewonyo et al., 2014, Consumer Financial Protection Bureau, 2014, Diamond et al., 2019, Ghosh et al., 2021, Grumbach and Sahn, 2020, Pierson et al., 2020, Zhang, 2018]. We note that discretization is not strictly necessary in all applications; Chen et al. [2019] and McCartan et al. [2023] present methods that utilize probabilistic outputs for disparity estimation, and DeLuca and Curiel [2022] sum probabilities directly when estimating the overall makeup of a population.

We are not the first to note that misclassification rates in BISG and related demographic imputation techniques are often unequal between races [Adjaye-Gbewonyo et al., 2014, Argyle and Barber, 2024, Baines

and Courchane, 2014, Elliott et al., 2009, Fiscella and Fremont, 2006], as well as correlated with other variables [Argyle and Barber, 2024]. However, these authors primarily focus on miscalibration and adjustments to the predictive models [Argyle and Barber, 2024], or tuning thresholds [Adjaye-Gbewonyo et al., 2014].

*Our contribution to this literature.* Our empirical results in Section 2 show that the discretization process (typically argmax or closely related threshold rules) leads to substantial bias in a commonly used commercial voter file, leading to a severe undercounting of voters of color – on top of the undercounting caused by continuous model miscalibration. We further find that a joint decision-making approach can eliminate discretization bias with negligible loss in individual-level accuracy. We thus caution against the use of existing discrete labels for sensitive applications where such bias would affect results. Instead, we recommend that (a) data vendors produce discretized labels using a joint decision-making approach, as we do; (b) when possible, consumers of voter files directly use the *continuous* scores instead of single discrete labels [Chen et al., 2019, DeLuca and Curiel, 2022, McCartan et al., 2023], or reconsider the use of imputed demographic data altogether. In Appendix C.1, we extend the analysis of Argyle and Barber [2024] to show that using continuous scores directly, or discretizing using our methods, outperforms simply improving the continuous model and then performing argmax discretization—the two sources of error (continuous miscalibration and discretization) are distinct, with distinct solutions.

# B Theoretical analysis

## B.1 Proofs

Recall that a classifier $q$ is **calibrated** when its continuous predictions are correct on average, $\Pr(Y = y|q(y,x) = c) = c$.[2] Calibration implies Bayesian consistency, i.e., the continuous model cumulatively assigns the proper mass to each class $y$: $\mathbb{E}_X[q(y,x)] = \Pr(y)$.

**Theorem 1.** *Consider calibrated classifier $q$ and the argmax decision rule $D_{argmax}$, with $N$ datapoints and $K$ classes, and $N > K$. Consider bias with respect to the aggregate posterior, $p_{ref} = p_{agg}$. Then, argmax bias is upper bounded by the predictive error of the classifier $q$:*

$$BIAS(y, D_{argmax}) \leq MAE(q). \tag{1}$$

*The bound is tight: there exist $F_{XY}$ and $q$ such that Equation (6) holds with equality for the plurality class.*

*Proof.*
For any continuous classifier $q$, denote the sets $S_a, S_b, S_c, S_d, S_e \subseteq \mathcal{X}$ as

$$x \in \begin{cases} S_a & \text{if } q(z,x) = 1, \\ S_b & \text{if } q(z,x) \in (\frac{1}{K}, 1) \\ S_c & \text{if } q(z,x) = \frac{1}{K}, \\ S_d & \text{if } q(z,x) \in (0, \frac{1}{K}) \\ S_e & \text{if } q(z,x) = 0 \end{cases}$$

with probability mass $\ell \triangleq \int_{x \in S_\ell} f(x)dx$ for $\ell = a, b, c, d, e$; that is, $a = \int_{x \in S_a} f(x)dx$, etc. Since the sets partition the entire probability space, we have $a + b + c + d + e = 1$.

For any class $y$, its amplification bias bias$(y)$ is maximized when the most data points have $y$ as its decision, and so we suppose the argmax decision rule breaks ties in favor of $z$, i.e., the decision rule outputs $z$ when $q(z,x) \geq q(y',x)$ for all $y' \in \mathcal{Y}$.

**We start with an example such that Equation (6) holds with equality.** Consider $q$ such that $b, d = 0$. Further suppose that for $x \in S_c$, we further have that $q(y,x) = \frac{1}{K}$, for all $y$. For $x \in S_e$, assume that there exists another class $y' \neq z$ such that $q(y',x) = 1$.

[2] For all $c$ of non-zero measure: $\forall c \in \left\{c : \left|\int_{(x,y)} \mathbb{I}[q(y,x) = c] f(x,y) d(y,x)\right| > 0\right\}$.

Recall that, by assumption, $q$ is consistent, i.e.,

$$p(z) = \mathbb{E}_X[q(z,x)] = \int_{x\in S_a} 1f(x)dx + \int_{x\in S_c} \frac{1}{K}f(x)dx + \int_{x\in S_e} 0f(x)dx \tag{2}$$
$$= a + \frac{c}{K}. \tag{3}$$

The key step is realizing that the *calibration* requirement on $q$ gives us a bound for MAE, and relates bias to classifier accuracy. Recall that calibration means that

$$\mathbb{E}_{XY}[Y = y | q(y,x) = \delta] = \delta \tag{4}$$

which implies that $\mathbb{E}_{XY}[Y = z | x \in S_a] = 1$, and $\mathbb{E}_{XY}[Y = z | x \in S_e] = 0$, and $\mathbb{E}_{XY}[Y = z | x \in S_c] = \frac{1}{K}$.

Equivalently, that $\int_{x\in S_a} f(x,z) = a$ and $\int_{x\in S_a} \sum_{y\neq z} f(x,y) = 0$. Similarly, $\int_{x\in S_e} f(x,z) = 0$, and $\int_{x\in S_c} f(x,z) = \frac{c}{K}$.

Then, we have

$$\text{MAE} = \int_x \sum_{y\in Y} f(x,y)|1 - q(y,x)|dx \tag{5}$$
$$= \int_x \left[ [f(x,z)|1 - q(z,x)|] + \left[ \sum_{y\neq z} f(x,y)|1 - q(y,x)| \right] \right] dx \tag{6}$$
$$= \int_{x\in S_a} \left[ [f(x,z)0] + \left[ \sum_{y\neq z} f(x,y)1 \right] \right] dx \qquad \text{defns of } S_\ell \tag{7}$$
$$+ \int_{x\in S_e} \left[ [f(x,z)1] + \left[ \sum_{y\notin\{z,y'\}} f(x,y)1 \right] + [f(x,y')0] \right] dx \tag{8}$$
$$+ \int_{x\in S_c} \left[ \left[ f(x,z)\frac{K-1}{K} \right] + \left[ \sum_{y\neq z} f(x,y)\frac{K-1}{K} \right] \right] dx \tag{9}$$
$$= 0 + 0 + c\left[\frac{K-1}{K}\right] \qquad \text{calibration implications} \tag{10}$$

Note that argmax (breaking ties in favor of $z$) assigns label $z$ for all $x \in S_a, S_c$. Thus, we have

$$p_{\text{argmax}}(z) = a + c. \tag{11}$$

Putting things together, we have

$$\begin{aligned}
\text{bias}(z) &= p_{\text{argmax}}(z) - p(z) \\
&= a + c - (a + \frac{c}{K}) = c\frac{K-1}{K} \\
&= \text{MAE}.
\end{aligned}$$

**Proof that the above is also an upper bound in Equation (6), that that is the worst case example.** Now we prove that this bound is the worst case for any calibrated (and thus consistent) $q_0$. The framework for the proof is, for any given setting (joint distribution $F_0$, calibrated and consistent $q_0$) and fixed class $z$, to transform the setting into the worst-case example above, where each transformation: (a) maintains the original marginal distribution $\Pr(y)$ of $F$; (b) either maintains or increases the mass of points

$x$ assigned to class $z$ (and thus either maintaining or increasing bias); and (c) does not increase MAE. Then, since the worst-case example satisfies the inequality, we have shown that the original example does as well.

Our proof below is notationally simpler with continuous $x \in \mathcal{X}$, such that there is no point mass ($F(\{x\}) = 0$, for all $x$). Thus, for discrete $\mathcal{X}$, we first transform it by adding a continuous feature dimension, where the corresponding feature $x_{\text{new dimension}} \in [0, 1]$ is distributed uniform at random, independent of the other features and $y$.

Recall that we assume that ties are broken consistently, with some ordering over the classes $y \in \mathcal{Y}$. Thus, we can consider the class $z$ such that any ties with any other class are broken in its favor: this class has the worst-case bias; for example for $F_0, q_0$ such that another class exhibits more bias, we can relabel the points (and so MAE is fixed) such that $z$ has the largest bias, at least as large as the original example. Thus, proving the result for $z$ is sufficient.

We start with the case for $K = 2$, i.e., binary decision-making, and then show how the proof extends generally. The idea is that for any $x \in S_b$ (i.e., $q_0(z, x) \in (\frac{1}{K}, 1)$), we can transform the example such that $x$ becomes in either $S_a$ or $S_c$ while maintaining the original class distribution $\Pr(y)$ and the fraction of points discretized to $z$, and preserving MAE. Similarly, for any $x \in S_d$, we can move $x$ to either $S_c$ or $S_e$ while maintaining consistency, increasing bias, and preserving MAE.

Step 1: Suppose mass $b$ of $S_b$ is positive, $b > 0$. Then, we move a set $S_{b\to a}$ to $S_a$ and a set $S_{b\to c}$ to $S_c$, respectively, maintaining consistency, where $S_{b\to a}$, $S_{b\to c}$ are a partition of $S_b$. In the binary example, we know that all $x$ are still discretized to $z$, since $q(z, x) > 1/2$ (and will remain so), and so $p_{\text{argmax}}$ is fixed.

Recall that consistency requires $p(z) = \mathbb{E}_X[q(z, x)] = \int_{x\in\mathcal{X}} q(z, x)f(x)dx$.

We can choose sets $S_{b\to a}$, $S_{b\to c}$ to maintain consistency and the class distribution of $z$ by satisfying the following:

$$\int_{x\in S_b} q_0(z, x)f(x)dx = \int_{x\in S_{b\to a}} 1f(x)dx + \int_{x\in S_{b\to c}} \frac{1}{K}f(x)dx \tag{12}$$

$$\iff \int_{x\in S_{b\to a}} q_0(z, x)f(x)dx + \int_{x\in S_{b\to c}} q_0(z, x)f(x)dx = \int_{x\in S_{b\to a}} 1f(x)dx + \int_{x\in S_{b\to c}} \frac{1}{K}f(x)dx \tag{13}$$

$$\iff \int_{x\in S_{b\to c}} (q_0(z, x) - \frac{1}{K})f(x)dx = \int_{x\in S_{b\to a}} (1 - q_0(z, x))f(x)dx \tag{14}$$

Note that such sets exist because $F_0$ is a continuous distribution in $\mathcal{X}$, and so expanding the set $S_{b\to c}$ monotonically and continuously increases the left-hand side, while monotonically and continuously decreasing the right-hand side.

Next, we claim that this transformation preserves MAE. As we used in Equation (10), calibration implies that the transformed error $\text{MAE}_1$ within the subset $S_{b\to a}$ is 0, and $\text{MAE}_1$ within the subset $S_{b\to c}$ is $F(S_{b\to c})\frac{K-1}{K}$.

Recall that $\text{MAE} = \sum_{y\in\mathcal{Y}} \int_{x\in\mathcal{X}} f(x, y)(1 - q(y, x))dx$. Then,

$$\text{MAE}_1 - \text{MAE}_0 \tag{15}$$

$$= \sum_{y\in\mathcal{Y}} \left[ \left[ \int_{S_{b\to c}} f(x, y) \left[ (1 - \frac{1}{K}) - (1 - q_0(y, x)) \right] dx \right] + \left[ \int_{S_{b\to a}} f(x, y) \left[ (1 - 1) - (1 - q_0(y, x)) \right] dx \right] \right] \tag{16}$$

$$= \sum_{y\in\mathcal{Y}} \left[ \left[ \int_{S_{b\to c}} f(x, y) \left[ q_0(y, x) - \frac{1}{K} \right] dx \right] - \left[ \int_{S_{b\to a}} f(x, y) \left[ 1 - q_0(y, x) \right] dx \right] \right] \tag{17}$$

$$= 0 \tag{18}$$

Where the last line follows from Equation (14) (the sets were chosen to maintain consistency, and this also maintains MAE). Note that Equation (14) only has this condition for the focal class $z$, whereas Equation (17) sums over classes. Note that, in the $K = 2$ example, maintaining consistency (and class distribution) of

one class automatically maintains it for the other class, and that the sets $\{x : q(z,x) \in (1/K, 1)\}$ and $\{x : q(y,x) \in (0, 1/K)\}$, $y \neq z$ are equivalent.

Thus $\text{MAE}_1 = \text{MAE}_0$ and $\text{bias}_1 = \text{bias}_0$.

Step 2: Similarly, now suppose mass $d$ of $S_d$ is positive, $d > 0$. Then, we move a set $S_{d\to c}$ to $S_c$ and a set $S_{d\to e}$ to $S_e$, respectively, maintaining consistency and MAE while *increasing* bias (in $S_d$, points are not discretized to $z$, but they are in $S_{d\to c}$ under the transformed example).

As in Step 1, choose the sets to maintain consistency and the class distribution of $z$ by:

$$\int_{x\in S_{d\to e}} (q_1(z,x) - 0) f(x)dx = \int_{x\in S_{d\to c}} (1/K - q_1(z,x)) f(x)dx$$

And then we have

$$\begin{aligned}
&\text{MAE}_2 - \text{MAE}_1 &(19)\\
&= \sum_{y\in\mathcal{Y}} \left[ \left[ \int_{S_{d\to e}} f(x,y) \left[ (1-0) - (1 - q_1(y,x)) \right] dx \right] + \left[ \int_{S_{d\to c}} f(x,y) \left[ (1 - \frac{1}{K}) - (1 - q_1(y,x)) \right] dx \right] \right] &(20)\\
&= \sum_{y\in\mathcal{Y}} \left[ \left[ \int_{S_{d\to e}} f(x,y) \left[ q_1(y,x) \right] dx \right] - \left[ \int_{S_{d\to c}} f(x,y) \left[ \frac{1}{K} - q_1(y,x) \right] dx \right] \right] &(21)\\
&= 0, &(22)
\end{aligned}$$

and so $\text{bias}_2 \geq \text{bias}_1$ and $\text{MAE}_2 = \text{MAE}_1$.

Thus, we have

$$\begin{aligned}
\text{bias}_0 &= \text{bias}_1 \leq \text{bias}_2 \\
&= \text{MAE}_2 \qquad \text{Equivalent to example for worst case where showed equality} \\
&= \text{MAE}_1 = \text{MAE}_0.
\end{aligned}$$

**Finally, we must show that the above argument holds for $K \geq 2$.** For the $K = 2$ case, the notation is simpler because transforming $q(z,x)$ also symmetrically transforms $q(y,x)$ for the class $y \neq z$, and that the sets $\{x : q(z,x) \in (1/K, 1)\}$ and $\{x : q(y,x) \in (0, 1/K)\}$ are equivalent. Thus, if $x \in S_{b\to c}$, then, simultaneously, we were moving $q(y,x)$ from less than $1/2$ to exactly $1/2$. This symmetry no longer holds for $z$ and a *fixed* $y \neq z$ because these factors depend on other classes as well (one can increase $q(z,x)$ while holding fixed $q(y,x)$). However, we show that we can still construct a sequence of transformations as follows.

Now, the strategy will be to partition the feature space $\mathcal{X}$ such that each partition can be converted to the worst-case example above, for some $k \leq K$ where only $k$ classes have non-zero probability in that partition. Then, since the identity holds within each partition, and MAE is a weighted average over the MAE within each partition (weighted by the partition mass), the result holds overall.

First, partition the feature space such that there are at most $K$ partitions, where the first partition has every class with non-zero probability, and the last partition only has one class with non-zero probability. Now, conduct the following transformations iteratively for each partition, that has $k = K, K-1, \ldots 1$ classes with non-zero probability.

- Define $S_a$, $S_e$ as before, where $q(z,x) = 1$ or $q(z,x) = 0$. Analogously, define $S_{b(k)}, S_{c(k)}, S_{d(k)}$ as:

$$x \in \begin{cases} S_{b(k)} & \text{if } q(z,x) \in (1/k, 1) \\ S_{c(k)} & \text{if } q(z,x) = 1/k, \\ S_{d(k)} & \text{if } q(z,x) \in (0, 1/k). \end{cases}$$

Finally, define $S_{b'}, S_{c'}, S_{d'}$ based on the maximum probability of a class $y \neq z$. Let $m(x) \triangleq \max_{y \neq z} q(y, x) \geq 1/k$. Then, let:

$$x \in \begin{cases} S_{b'} & \text{if } q(z, x) \in (m(x), 1) \\ S_{c'} & \text{if } q(z, x) = m(x), \\ S_{d'} & \text{if } q(z, x) \in (0, m(x)) \end{cases}$$

- While the mass of set $S_{b'}$ within the partition is more than zero and there are $k$ classes with non-zero probability, note that for each $x \in S_{b'}$, there exists *at least one* class $y' \neq z$ such that $q(y', x) \in (0, 1/k)$. Now, we transform similarly to the binary case. We will move $q(y', x)$ toward 0 or $1/k$, to maintain consistency and $\sum_y q(y, x) = 1$ for all $x$, for each $x$ for which we change $q(z, x)$. For some $x$, this involves moving $x$ to $S_a$ ($q(z, x) = 1$) as before. For other $x$, we will move to $S_{b'}$, balancing these moves to maintain consistency and MAE as before. Note that the transformations maintain $\text{bias}(z)$.
- Now, analogously, move $x \in S_{d'}$ to either $S_e$ or $S_{c'}$, while balancing the changes in $q(z, x)$ with corresponding changes in $q(y', x)$ for the $y' = \arg\max_y q(y, x)$. These transformations either maintain or increase $\text{bias}(z)$.
- Note that these transformations will continue until for $x$ originally in this partition, either: (1) there are now less than $k$ classes $y$ for which $q(y, x) > 0$; (2) we have $x \in S_a \cup S_{c(k)} \cup S_e$: the key is noting that, due to these transformations, the max probability values $m(x)$ also become $1/k$, for points that remain in this partition where there are $k$ classes with non-zero probability.

Finally, note that we have converted the original setting, while maintaining MAE and not decreasing $\text{bias}(z)$, to a partition such that within each partition, the classifier behaves according to the worst-case example for some $k$, and so bias is bounded by the MAE within that partition. The overall bound follows from noting that both overall BIAS and overall MAE are weighted averages of the BIAS and MAE within each partition, weighted by the partition mass. □

**Corollary 1.** *Suppose $x$ provides no information and $q(y, x) = \Pr(y)$ for all $x, y$. Then, with argmax discretization, bias with respect to the aggregate posterior $p_{agg}$ is maximized and all data points are classified as a plurality class:*

$$BIAS(y, D_{argmax}) = \begin{cases} 1 - \Pr(y), & \text{if } y = \arg\max_w p(w), \\ -\Pr(y), & \text{otherwise.} \end{cases}$$

*Proof.* The result immediately follows by definition of argmax. For the argmax class $z$, all points are classified to it and so $\hat{p}_{\text{marg}}(\cdot, z) = 1$. Then $\text{MAE} = 1 - p_{\text{ref}}(z)$ and $\text{bias}(z, \cdot, \cdot) = 1 - p_{\text{ref}}(z)$ for any $\{x_i\}$. □

**Corollary 2.** *Suppose the classifier is perfect, i.e., $\forall (x, y) \sim F_{XY}$, we have $q(z, x) = \begin{cases} 1, & \text{if } z = y, \\ 0, & \text{otherwise.} \end{cases}$ Then, there is no amplification bias with respect to the prior or aggregate posterior; $BIAS(y, D_{argmax}) = 0$.*

*Proof.* We wish to show not only that bias $\leq 0$ but bias $= 0$ exactly. The result immediately follows from the definition of the aggregate posterior or the prior. Due to perfect prediction, we have that $\mathbb{E}\left[\hat{p}_{\text{marg}}(\cdot, y)\right] = \Pr(y)$, for all $y$. With a consistent classifier (implied by our notion of calibration), we have that

$$\mathbb{E}[p_{\text{agg}}(y, \{x_i\})] \triangleq \mathbb{E}\left[\frac{1}{N} \sum_i q(y, x_i)\right] = \Pr(y).$$

□

Finally, we note that Theorem 1 immediately also implies a lower bound on negative bias,

$$\mathrm{BIAS}(y) \geq -(K-1)\mathrm{MAE}(q),$$

because the negative bias of a class is bounded by the sum of positive biases for the remaining $K-1$ classes. We conjecture that it is possible to tighten this bound, with an analogous proof as that of Theorem 1.

**Theorem 2.** *Consider Bayes optimal $q$, and $N > K$. For all joint distributions $F$:*

*(i). For every $\gamma$, $p_{ref}$ there exists a joint decision-making rule that maximizes $O^{\gamma}(D, q, p_{ref})$ in Equation (5).*

*(ii). The argmax decision rule $D_{argmax}$ maximizes $O^1(D, q, p_{ref})$, i.e., is accuracy maximizing.*

*(iii). $D_{argmax}$ is the only Pareto optimal independent decision rule for non-trivial $p_{ref} \in \mathcal{P}$. No independent decision rule $D$ maximizes objective $O^{\gamma}$ for any $\gamma$, unless $\gamma = 1$ and $D$ agrees with $D_{argmax}$ with probability 1.*

*Proof.* **Proof for Part (i)** The result immediately holds for joint decision-making rules, by construction. Consider the multi-objective optimization decision rules defined in (1):

$$D^{\gamma}(\{q(x_i)\}, p_{\mathrm{ref}}) = \arg\max_{\{\hat{y}_i\}} \gamma \left( \frac{1}{N} \sum_{i=1}^{N} q(\hat{y}_i, x_i) \right) + (1-\gamma)\mathrm{fid}(p_{\mathrm{ref}}, \hat{y}_{1:N}).$$

Now, note that the objective can be decomposed using the tower property, as follows:

$$\begin{aligned}
O_N^{\gamma}(D, q, p_{\mathrm{ref}}) &= \gamma \mathrm{ACC}_N(D, q) + (1-\gamma)\mathrm{FID}_N(D, q, p_{\mathrm{ref}}) \\
&= \mathbb{E}_{F_{\{X\}}} \left[ \mathbb{E}_{F_{\{Y\}|\{X\}}} \left[ \gamma \mathrm{acc}(y_i, D(\{q(x_i)\})) + (1-\gamma)\mathrm{fid}(p_{\mathrm{ref}}, D(\{q(x_i)\})) \mid \{X_i\} = \{x_i\} \right] \right] \\
&= \mathbb{E}_{F_{\{X\}}} \left[ \mathbb{E}_{F_{\{Y\}|\{X\}}} \left[ \gamma \frac{1}{N} \sum_{i}^{N} \mathbb{I}\left[ D(\{q(x_i)\}) = y_i \right] + (1-\gamma)\mathrm{fid}(p_{\mathrm{ref}}, D(\{q(x_i)\})) \mid \{X_i\} = \{x_i\} \right] \right] \\
&= \mathbb{E}_{F_{\{X\}}} \left[ \mathbb{E}_{F_{\{Y\}|\{X\}}} \left[ \gamma \frac{1}{N} \sum_{i}^{N} q(D(\{q(x_j)\})_i, x_i) + (1-\gamma)\mathrm{fid}(p_{\mathrm{ref}}, D(\{q(x_i)\})) \mid \{X_i\} = \{x_i\} \right] \right]
\end{aligned}$$

Where the last line follows from, given Bayes optimal $q$, we have for all $\hat{y}_i$

$$q(\hat{y}_i, x_i) = \mathbb{E}_{F_{Y|X}}[Y = \hat{y}_i | X = x_i].$$

Thus, the optimization decision rule maximizes the inner expectation in the final line, for each given dataset $\{x_i\}$. Thus, the corresponding rule maximizes $O_N^{\gamma}(D, q, p_{\mathrm{ref}})$, as desired.

**Proof for Part (ii)** The result follows directly from the classifier being Bayes optimal – for each row, you cannot do better than picking the most likely class for that row.

Recall that $D(\{q(x_i)\})$ refers to the set of decisions $\hat{y}_{1:N}$ with dataset $x_{1:N}$, where the decisions can be jointly made across data points. Note that, for the Bayes optimal classifier $q^*$, we have

$$\mathbb{E}_{F_{Y|X}}\left[\mathbb{I}\left[Y = y\right] \mid X = x\right] = q^*(y, x)$$

Thus, we have:

$$
\begin{aligned}
O_N^1(D, q^*, \cdot) &= \mathrm{ACC}_N(D, q^*) && (23)\\
&= \mathbb{E}_F\left[\mathrm{acc}(y_{1:N}, D(\{q^*(x_i)\}))\right] && (24)\\
&= \mathbb{E}_F\left[\frac{1}{N}\sum_i \mathbb{I}\left[\hat{y}_i = y\right]\right] && \text{let } \hat{y}_i \triangleq D(\{q^*(x_j)\}_{j=1}^N)_i \quad (25)\\
&= \mathbb{E}_{F_{\{X\}}}\left[\mathbb{E}_{F_{\{Y\}|\{X\}}}\left[\frac{1}{N}\sum_i \mathbb{I}\left[Y_i = \hat{y}_i\right] \mid \{X_i\} = \{x_i\}\right]\right] && \text{tower property} \quad (26)\\
&= \mathbb{E}_{F_{\{X\}}}\left[\frac{1}{N}\sum_i \mathbb{E}_{F_{Y|X}}\left[\mathbb{I}\left[Y = \hat{y}_i\right] \mid X = x_i\right]\right] && \text{independent sampling of datapoints} \quad (27)\\
&= \mathbb{E}_{F_{\{X\}}}\left[\frac{1}{N}\sum_i q^*(\hat{y}_i, x_i)\right] && (28)\\
&\leq \mathbb{E}_{F_{\{X\}}}\left[\frac{1}{N}\sum_i q^*(\arg\max_y q^*(y, x_i), x_i)\right] && (29)\\
&\triangleq O_N^1(D_{\mathrm{argmax}}, q^*, \cdot). && (30)
\end{aligned}
$$

**Proof for Part (iii)**. The proof for Part (iii) is more involved. Note that both components of the objective are non-negative, and from Part (i) we have examples of joint decision rules that are optimal for every data sample $\{x_i\}$. Thus, to prove that a rule is not Pareto optimal for a given $F$, it is sufficient to show the set of dataset samples $\{x_i\} \sim F_X$ for which the rule is not optimal for that sample has positive probability. Since we are considering independent rules and a fixed $F, q$, we will denote $D(q(x))$ as the decision for datapoint $x$ for notational simplicity.

**Binary setting, K = 2**. For clarity of exposition, we first prove the result in detail for a binary classification setting where $y \in \{0, 1\}$, and then extend the proof for all $K$. Consider independent decision rule $D$ that is not identical to the argmax rule.

**Case 1. Non-monotonic or random rules.** If a rule, with positive probability, is such that $q(1, x_1) > q(1, x_2)$ but $D(q(x_2)) = 1$ and $D(q(x_1)) = 0$ , then it is suboptimal with positive probability. There exists some $D'$ where $D'(x) = D(x)$ for all $x$ except that $D(x_1) = 1$ and $D(x_2) = 0$. Then the marginal distribution $p_{\mathrm{marg}}$ remains the same and the expected accuracy of $D'$ is greater than that of $D$.

**Case 2. Threshold rules.** Thus, it is sufficient to consider rules with a threshold $p$, where $D_p(q(x)) = 1$ if and only if $q(1, x) \geq p$, and 0 otherwise. Note that $p = 0.5$ corresponds to the argmax decision rule. We now prove that for any other $p \neq 0.5$, with positive probability there exists a test sample such that the rule is suboptimal. Without loss of generality, assume $p < .5$, and a similar proof holds for $p > .5$.

By assumption, we have that $\exists \delta > 0$ such that $D_p$ disagrees with the argmax rule with positive probability $\delta$, i.e., $\Pr(q(1, x) \in (p, .5)) = \delta$. Then, with probability equal to $\delta^N > 0$, we have that the *entire* data sample $x_{1:N}$ is such that $q(1, x_i) \in (p, .5)$, and so $D_p$ (which has $\hat{y}_i = 1, \forall i$) disagrees with the argmax rule on *every* datapoint (which has $\hat{y}_i = 0, \forall i$). By the assumption of non-trivial reference distribution $p_{\mathrm{ref}} \in \mathcal{P}_N$, we have

$$p_{\mathrm{ref}}(0, \{x_i\}) \geq \frac{1}{N}$$

and so we can improve both accuracy and distributional fidelity by switching a decision on at least one point from $\hat{y}_i = 1$ to $\hat{y}_i = 0$.

**Extending argument for all $K \geq 2$.** Now, we show that the argument holds for any $K$, for any $F, q$ – either an independent decision rule $D$ is equivalent to the argmax rule (with probability 1, they make the same decisions), or it is not on the Pareto curve. The argument is similar to the above, and is as follows.

Consider a class $y$ such that there exists a set $X \subseteq \mathcal{X}$ with $F(X) > 0$ such that, for $x \in X$, the argmax rule discretizes $x$ to $y$ but the rule $D$ does not: $D_{\text{argmax}}(q(x)) = y$, but $D(q(x)) \neq y$. Such a class $y$ and set $X$ exists since $D$ is not equivalent to $D_{\text{argmax}}$.

Now, consider a dataset sample such that, $x_i \in X$ for all $i$. This happens with positive probability $F(X)^N$. For this sample, $D$ disagrees with $D_{\text{argmax}}$ on *every* data point $x_i$, and note that $D_{\text{argmax}}(q(x_i)) = y$ for all $x_i$. By the assumption of non-trivial reference distribution $p_{\text{ref}} \in \mathcal{P}_N$, we have

$$p_{\text{ref}}(y, \{x_i\}) \geq \frac{1}{N},$$

i.e., matching the reference distribution would allocate at least one data point to $y$. Thus, we can improve both accuracy and fidelity to the reference distribution by switching the decision of at least one point to $y$ (in particular, finding a class $y'$ that is over-allocated compared to the reference distribution).

□

## B.2 Simulating the Pareto Curve of Methods

Figure S1 demonstrates Theorem 2 with a simulated, Bayes optimal predictor. Data is generated as described in Section 5, using $\sigma = 0.5$ (MAE = 0.42).

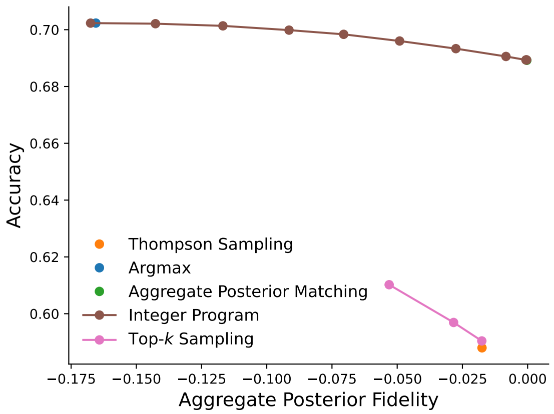


Figure S1: In simulation, the performance of various commonly used decision rules. The results illustrate Theorem 2: the argmax rule maximizes accuracy, but is the only Pareto optimal independent rule. Simulation details and decision rules are described in Section 5.

## B.3 Simulating Worst-Case Bias

We now draw data according to the worst-case example discussed in the proof of Theorem 1. Figure S2 replicates Figure 4a from the main text, with this alternative synthetic data approach. Note that the bias of the most common class is equal to MAE, as we prove in Theorem 1. Class 1 corresponds to the plurality class $z$, while the other classes all have equal probability. In the notation of our proof, we have $e = 0$ and $a = 1 - c$, and we vary $c$ in the plot.

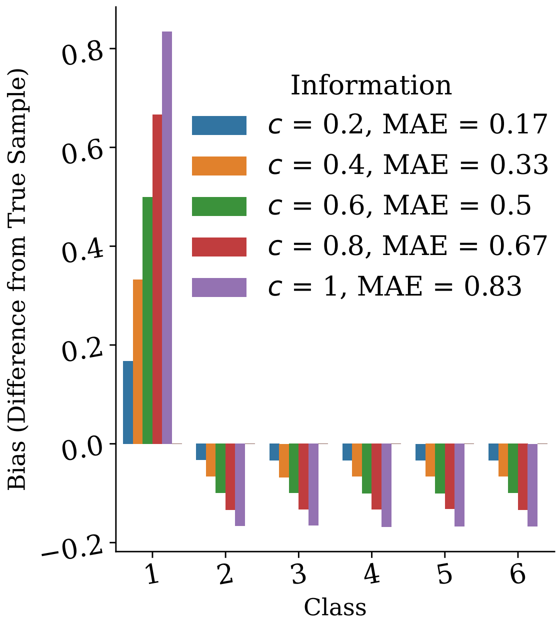


Figure S2: Simulated bias for each class for the worst-case example in which bias of the most common class is equal to the MAE.

# C Additional empirical analyses

## C.1 Implications in Downstream Analyses: Voter Turnout

We now show how the choice of discretization methods (and whether to discretize at all) might affect downstream analyses.

Background. We build on an analysis by Argyle and Barber [2024] examining voter turnout by predicted race and income—they show that using BISG (discretized the standard way using argmax) leads to implausible (and incorrect, compared to ground-truth) conclusions: for Black people, voter turnout *falls* as the median income of the census tract increases, to below 10%.

Argyle and Barber [2024] proposed to decrease such biases (and disparate false positives/negative rates by group) via a machine learning approach: they augment the probability outputs of a BISG variant [Imai and Khanna, 2016] with additional information (e.g., party affiliation, sex, age, the racial makeup of the citizen voting age population of the neighborhood, etc.) to train a random forest. This random forest model has a weighted objective function by class, to more strongly penalize errors for smaller groups. Their random forest approach directly outputs discrete labels (internally, via argmax), and so can be viewed as the following two-step process, in the language of our work: they (1) *improve the continuous BISG model by adding additional features for each individual and using a random forest model, weighted by class*; (2) apply *argmax discretization* to the improved continuous model scores.

Our analysis. We extend the analysis of Argyle and Barber [2024] to illustrate the lessons of our work, with respect to how it affects downstream outcomes. Using continuous random forest scores akin to their model, we measure voter turnout by racial group as a function of geographic income level, using both the continuous scores directly and the various discretization approaches. The methods analyzed are summarized in Table S1. In short, we use as a base model the continuous scores from either the BISG model (provided by the replication file of Argyle and Barber [2024]), or those outputted by a random forest model (retrained by us; designed to replicate the approach of Argyle and Barber [2024] using their data). Then, we conduct the downstream analyses using either the continuous scores directly, or discretized according to various methods.

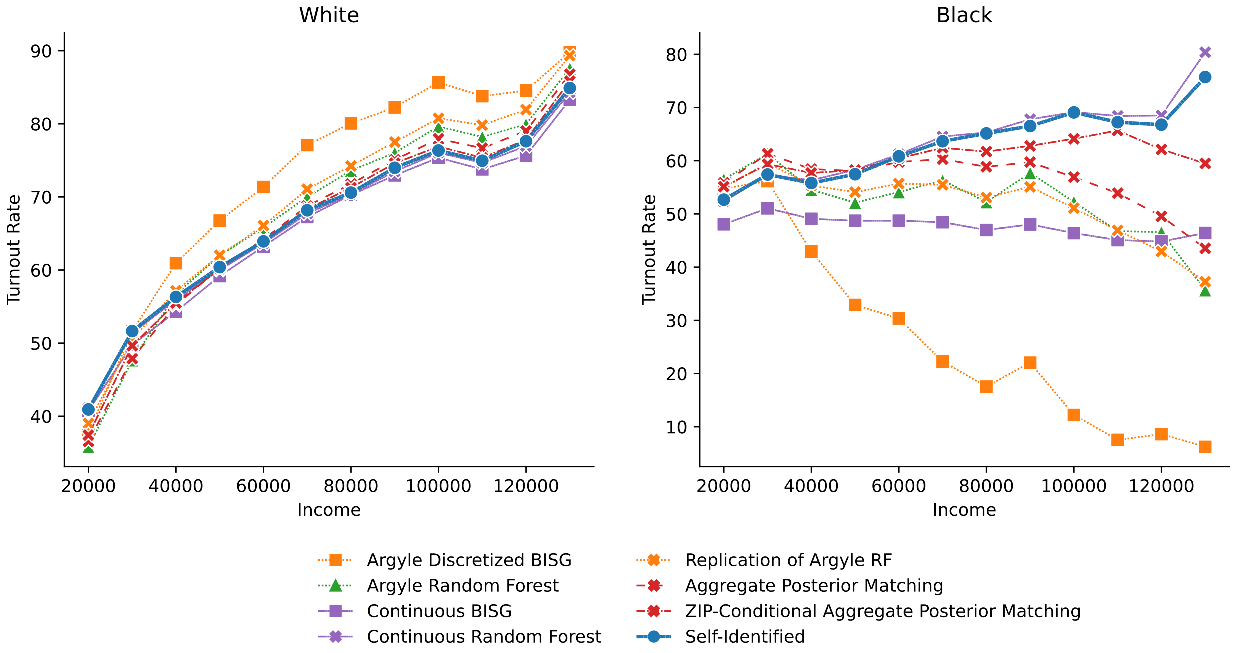


Figure S3: Turnout rates among *White* (left) and *Black* (right) voters as estimated by various combinations of continuous model and discretization method. Of all discretization methods, matching conditioned on ZIP code most closely matches the self-identified ground truth (Blue circle). Of all estimation methods, using the continuous estimator with the replication random forest probabilities performs best, but even BISG performance substantially improves when using the continuous estimator over argmax. Note that the replication random forest outputs under argmax closely map onto the discrete Argyle random forest outputs.

Findings overview. Figure S3 illustrates turnout rates as estimated by each of the approaches for *White* and *Black* voters, respectively, as a function of geographic level income. In each figure, the Blue (circle) line illustrates estimated voter turnout when self-report data in the voter file is used, which we will use as ground truth. The patterns illustrate the lessons of our work:

- **Continuous scores should be used when possible for downstream analyses, to avoid discretization bias.** Notably, when the continuous random forest (from our replication model) are used directly, the estimates closely mimic ground truth: the Purple (cross) line is almost identical to the Blue (circle) line. More generally, for each of the BISG and Random Forest models, using the continuous scores leads to more accurate estimates than any does discretization of those scores.
- **Improving the continuous scores helps, but argmax discretization bias remains even with accurate but imperfect models.** Notably, the approach of Argyle and Barber [2024] to add additional data does lead to a substantially improved predictive model and decreases error when argmax discretization is used. The Green (triangle) line of is much closer to ground truth than the Orange (square) line, even though both use argmax discretization. However, as reported by Argyle and Barber [2024] and replicated here, there is still a substantial gap between the argmax discretized random forest and ground truth.
- **If the use of discretization is necessary, our joint optimization approaches decrease errors in downstream analyses, without using additional information.** Finally, our discretization approaches (in Red) are substantially closer to the estimates using self-identified ground truth than is argmax discretization of the same underlying random forest in Green (triangle), which is designed to

| | Underlying Model | Discretization Method | Model Training Data |
|---|---|---|---|
| Argyle Random Forest (RF) | Discrete RF | Argmax (Equivalent) | Florida |
| Argmax Discretized BISG | BISG | Argmax | |
| Continuous BISG | BISG | Continuous | |
| Argmax Discretized Replication RF | Replication RF | Argmax | N. Carolina |
| AP Matching | Replication RF | Agg. Posterior Matching | N. Carolina |
| ZIP-Conditional AP Matching | Replication RF | ZIP-Conditional AP | N. Carolina |
| Continuous Replication RF | Replication RF | Continuous | N. Carolina |

Table S1: The models and data used to generate predictions in our replication and extension of Argyle and Barber [2024] in Appendix C.1.

replicate the proposed solution of Argyle and Barber [2024] (in Orange (cross)). Notably, they do not use any additional information than is available to train the underlying continuous model, and so is an improvement "for free."

Overall, the results (along with those of Argyle and Barber [2024]) speak to the importance of racial/ethnic mis-labeling in affecting important downstream analyses.

Methodological details. We directly use the data provided in the replication file of Argyle and Barber [2024], for North Carolina. Their replication file contains their (discrete) model outputs for each individual in their North Carolina file, as well as continuous BISG scores. As their random forest model only outputs discrete label, we use their replication data [Barber and Argyle, 2023] to train a probabilistic random forest using similar parameters (including class weights) that outputs continuous predictions.[3]

After training our random forest replication model, we calculate labels using each method for the test set (half of) North Carolina voter file. Then, we calculate voter turnout mimicking the method of Argyle and Barber [2024], dividing the counted turnout by group by the citizen voting age population per race (a known, fixed number) and income bracket. For the continuous model analyses, each voter who turned out contributes a fraction to the numerator based on their predicted probability for belonging to that group. For example, a voter who is predicted to be *Black* with 60% probability and *White* with 35% would contribute 0.6 to the numerator for *Black* turnout and .35 to that for *White* turnout. Note that since we count turnout using only half the voter file, we then scale vote counts by a factor of 2.

## C.2 Population Proportions, Error Rates, and Model calibration

In North Carolina, Table S2 shows the population size (normalized by both overall population count and true count for that group) for each group, and Table S3 contains the false positive and false negative rates—each when using different discretization methods to discretize the continuous commercial model scores. Like others have previously observed [Adjaye-Gbewonyo et al., 2014, Argyle and Barber, 2024, Imai and Khanna, 2016, Imai et al., 2022], error rates differ between classes; the *Caucasian* class has far more false positives, while populations of color have more false negatives, reflecting a predicted population that is disproportionately white. Error rate disparities are not just a function of bias; notably, while Thompson sampling creates little to no bias, the loss in accuracy exacerbates both kind of error.

[3]Note that this is not an exact replication; to our understanding, the random forest training data in Florida is not available. To substitute, we divide their North Carolina data ($N$ = 6,667,100 after cleaning) into a 50-50 train–test split. Our results are calculated on the test sample ($N$ = 3,333,550). The purpose of our replication is not to build a competitive continuous model, but the most analogous continuous equivalent of their discrete random forest. While their model predictions on North Carolina are out-of-distribution (trained using Florida data), we compare the argmax outputs of this continuous replica and find the performance to be comparable, as evidenced by the Green (triangle) and Orange (cross) lines being similar in Figure S3. To further validate our comparison, we discretize the probability outputs from our replication random forest with argmax and find similar performance to their original random forest, with minor improvements (accuracy to .866 from .853, macro-averaged F1 score from to .612 from .588) that likely arise from training on in-sample data.

The tables further shows that continuous model miscalibration and discretization bias are distinct phenomena. For the *African-American* group, the continuous scores are negatively biased (predicts lower probabilities than a calibrated classifier would), and this is exacerbated by discretization. For the *Asian* group, on the other hand, the continuous scores are *positively* biased (predicts higher probabilities, potentially because of characteristics of the distribution of Asian last names compared to last name distributions of other groups), but that argmax discretization leads to *substantially* fewer overall people being labeled as *Asian* (partially because the group is comparatively small).

| Method | African-American | Hispanic | Asian | Native American | Caucasian |
|---|---|---|---|---|---|
| True Population | 0.227 | 0.035 | 0.016 | 0.008 | 0.714 |
| Aggregate Posterior of Model | 0.190 | 0.035 | 0.018 | 0.013 | 0.744 |
| Threshold at 0.8 (Among 67.9% Not Dropped) | 0.080 | 0.018 | 0.010 | 0.002 | 0.890 |
| Integer Program, $\gamma = 0.9$ | 0.190 | 0.035 | 0.015 | 0.006 | 0.753 |
| Commercial (Argmax) | 0.163 | 0.030 | 0.013 | 0.005 | 0.789 |
| Aggregate Posterior Matching | 0.190 | 0.035 | 0.018 | 0.013 | 0.744 |
| Data-Driven Threshold Matching Heuristic | 0.190 | 0.036 | 0.018 | 0.012 | 0.744 |
| County-Conditional Aggregate Posterior Matching | 0.190 | 0.035 | 0.018 | 0.013 | 0.744 |
| Thompson Sampling | 0.190 | 0.035 | 0.018 | 0.013 | 0.744 |
| Top-2 Sampling | 0.190 | 0.033 | 0.015 | 0.007 | 0.755 |
| True Population Matching | 0.227 | 0.035 | 0.016 | 0.008 | 0.714 |
| County-Conditional True Population Matching | 0.227 | 0.035 | 0.016 | 0.008 | 0.714 |

(a) Fractions of the total population.

| Method | African-American | Hispanic | Asian | Native American | Caucasian |
|---|---|---|---|---|---|
| True Population | 1.000 | 1.000 | 1.000 | 1.000 | 1.000 |
| Aggregate Posterior of Model | 0.836 | 1.017 | 1.108 | 1.644 | 1.042 |
| Threshold at 0.8 (Among 67.9% Not Dropped) | 0.351 | 0.506 | 0.653 | 0.264 | 1.246 |
| Commercial (Argmax) | 0.718 | 0.851 | 0.799 | 0.677 | 1.105 |
| Integer Program, $\gamma = 0.9$ | 0.836 | 1.013 | 0.957 | 0.786 | 1.055 |
| Aggregate Posterior Matching | 0.836 | 1.017 | 1.108 | 1.644 | 1.042 |
| Data-Driven Threshold Matching Heuristic | 0.837 | 1.026 | 1.129 | 1.500 | 1.042 |
| County-Conditional Aggregate Posterior Matching | 0.836 | 1.017 | 1.108 | 1.644 | 1.042 |
| Thompson Sampling | 0.836 | 1.018 | 1.106 | 1.637 | 1.042 |
| Top-2 Sampling | 0.838 | 0.938 | 0.957 | 0.866 | 1.057 |
| True Population Matching | 1.000 | 1.001 | 1.002 | 1.002 | 1.000 |
| County-Conditional True Population Matching | 1.000 | 1.000 | 1.001 | 0.999 | 1.000 |

(b) Fractions of the true population of each group.

Table S2: The racial/ethnic makeup of the North Carolina population according to different discretization methods. In Table S2a, bias is calculated as the difference between discretized rows and the True Population or Aggregate Posterior rows. In Table S2b, such differences provide the percentage changes in Section 2.

Figure S4 shows the calibration curve of the voter file's continuous scores, assessed in North Carolina for individuals for whom self-reported ground truth data is available.

| Method | African-American | Hispanic | Asian | Native American | Caucasian |
|---|---|---|---|---|---|
| Threshold at 0.8 (Among 67.9% Not Dropped) | 0.008 | 0.003 | 0.001 | 0.000 | 0.376 |
| Integer Program, $\gamma = 0.9$ | 0.057 | 0.011 | 0.005 | 0.002 | 0.322 |
| Commercial (Argmax) | 0.041 | 0.007 | 0.003 | 0.001 | 0.392 |
| Aggregate Posterior Matching | 0.057 | 0.011 | 0.007 | 0.009 | 0.308 |
| Data-Driven Threshold Matching Heuristic | 0.057 | 0.011 | 0.008 | 0.007 | 0.309 |
| County-Conditional Aggregate Posterior Matching | 0.061 | 0.011 | 0.008 | 0.009 | 0.321 |
| Thompson Sampling | 0.090 | 0.017 | 0.009 | 0.010 | 0.416 |
| Top-2 Sampling | 0.088 | 0.013 | 0.007 | 0.004 | 0.425 |
| True Population Matching | 0.084 | 0.011 | 0.006 | 0.004 | 0.260 |
| County-Conditional True Population Matching | 0.085 | 0.011 | 0.006 | 0.004 | 0.262 |

(a) False Positive Rates in NC.

| Method | African-American | Hispanic | Asian | Native American | Caucasian |
|---|---|---|---|---|---|
| Threshold at 0.8 (Among 67.9% Not Dropped) | 0.405 | 0.312 | 0.304 | 0.594 | 0.010 |
| Integer Program, $\gamma = 0.9$ | 0.359 | 0.292 | 0.365 | 0.467 | 0.074 |
| Commercial (Argmax) | 0.423 | 0.351 | 0.395 | 0.495 | 0.052 |
| Aggregate Posterior Matching | 0.359 | 0.294 | 0.348 | 0.407 | 0.082 |
| Data-Driven Threshold Matching Heuristic | 0.359 | 0.292 | 0.344 | 0.413 | 0.082 |
| County-Conditional Aggregate Posterior Matching | 0.371 | 0.295 | 0.353 | 0.501 | 0.087 |
| Thompson Sampling | 0.469 | 0.458 | 0.453 | 0.593 | 0.125 |
| Top-2 Sampling | 0.462 | 0.435 | 0.444 | 0.588 | 0.113 |
| True Population Matching | 0.286 | 0.292 | 0.360 | 0.445 | 0.104 |
| County-Conditional True Population Matching | 0.289 | 0.292 | 0.361 | 0.471 | 0.105 |

(b) False Negative Rates in NC.

Table S3: Error rates vary between discretization methods. Note that matching- and integer program-based solutions even out these rates, while Pareto-dominated sampling-based approaches worsen both kinds of errors.

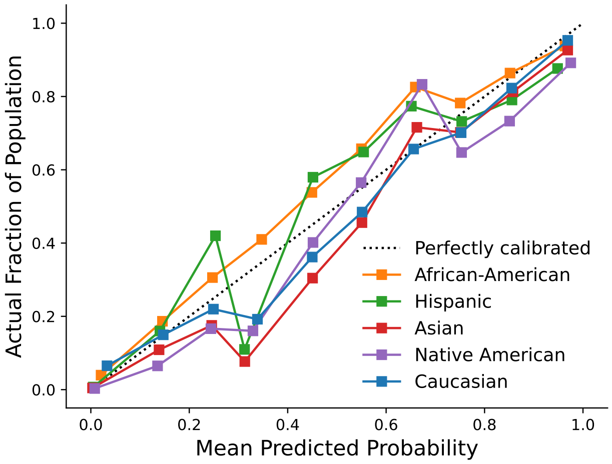


Figure S4: Calibration plot for the continuous scores in the voter file, assessed using North Carolina individuals for whom self-reported ground truth data is available. Mathematically, the $x$ axis corresponds to (binned) $q(y, x)$, and the $y$ axis corresponds to $\mathbb{E}[Y = y|q(y, x) = c]$. Thus, a perfectly calibrated classifier would be on the $y = x$ line for each group. Note that *African-American* is fully above the line, meaning that for any predicted fraction $c$, a *higher* proportion of the population is actually *African-American*. Thus, the classifier is under-predicting the group, leading to even the aggregate posterior under-counting the group in Figure 2a. Conversely, *Caucasian* is primarily below the line, indicating that the continuous model is over-predicting the group.

## C.3 Additional discretization methods

As described in Section 5, we empirically examine and plot additional discretization methods, such as Top-$k$ sampling. Results are in Figure S5 and table S4. These methods are also included in our replication analyses in Appendices C.4 to C.6.

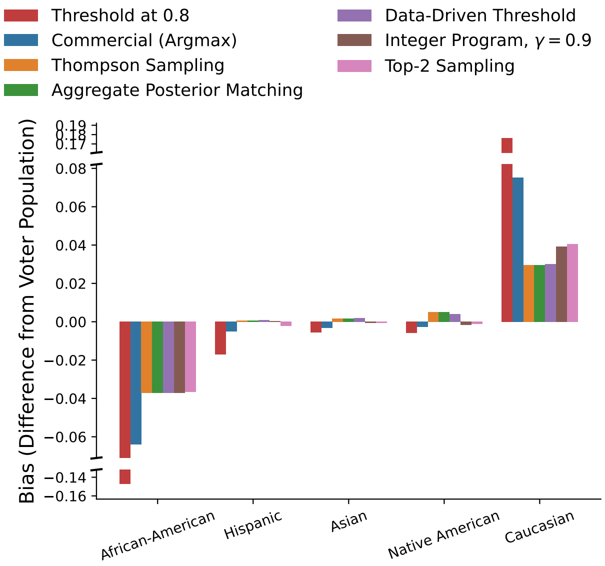


Figure S5: Extension of Figure 2a with additional decision-making rules plotted.

| | Accuracy with Ground Truth | Fidelity to Ground Truth | Fidelity to Aggregate Posterior |
|---|---|---|---|
| Threshold at 0.8 (Among 67.9% Not Dropped) | **0.928** | -0.352 | -0.293 |
| Integer Program, $\gamma = 0.9$ | 0.846 | -0.079 | -0.019 |
| Commercial (Argmax) | 0.844 | -0.150 | -0.091 |
| Aggregate Posterior Matching | 0.841 | -0.074 | **-0.000** |
| Data-Driven Threshold Matching Heuristic | 0.841 | -0.074 | -0.002 |
| County-Conditional Aggregate Posterior Matching | 0.834 | -0.074 | -0.000 |
| Thompson Sampling | 0.776 | -0.074 | -0.000 |
| Top-2 Sampling | 0.787 | -0.081 | -0.023 |
| Ground Truth Marginal Matching | 0.841 | -0.000 | -0.074 |
| County-Conditional Ground Truth Marginal Matching | 0.840 | **-0.000** | -0.074 |

Table S4: Extension of Table 1 with additional decision-making rules plotted. Note that access to the ground truth distribution makes bias minimization trivial, while achieving accuracy comparable to the aggregate posterior.

## C.4 Replication on Public Data

We replicate our analysis and results from publicly available data in North Carolina on imputation models developed by Greengard and Gelman [2024], whose data and code are publicly available for replication [Greengard and Gelman, 2023]. Their data in North Carolina ($N = 4{,}233{,}012$) uses $K = 6$ race/ethnicity categories: (non-Hispanic) *Black*, *American Indian and Alaskan Native (AIAN)*, *Asian and Pacific Islander (API)*, *White*, and *Hispanic*, with *Hispanic* encompassing voters of any racial background.

We examine three sets of models and predicted probabilities used by Greengard and Gelman [2024]: two implementations of Bayesian Improved Surname Geocoding (BISG) (the standard race prediction algorithm), as well their novel contribution, a raking-based improved model. Their two implementations of BISG use information from voter registration and from the US Census.

Figure S6 shows calibration plots for the two models provided; BISG on the voter file in particular is approximately calibrated for both *White* and *Black* groups, but not for other groups. In Figure S7, we observe that argmax bias persists. Notably, even when aggregate probabilities may overrepresent *Black* voters and underrepresent *White*, argmax discretization *still* leads to undercounting for the former and overcounting for the latter. However, because the Raking predictive model comparatively under-predicts *White* individuals, all discretization approaches that use the aggregate posterior under-count the *White* class. Figure S8 shows that, with more calibrated models, argmax is accuracy-maximizing over joint rules, more closely resembling the Bayes optimal setting in simulation Figure S1. Lastly, Figure S9 replicates Figure 3.

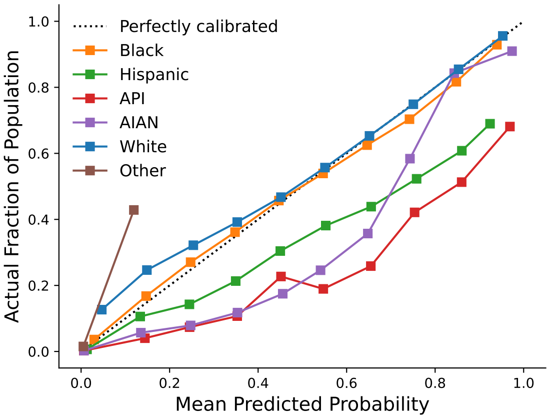


(a) Voterfile BISG.

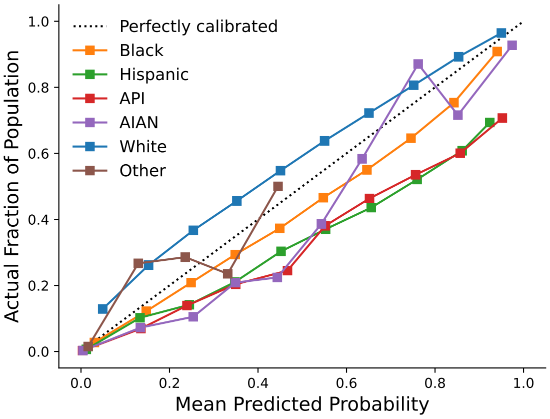


(b) Census BISG.

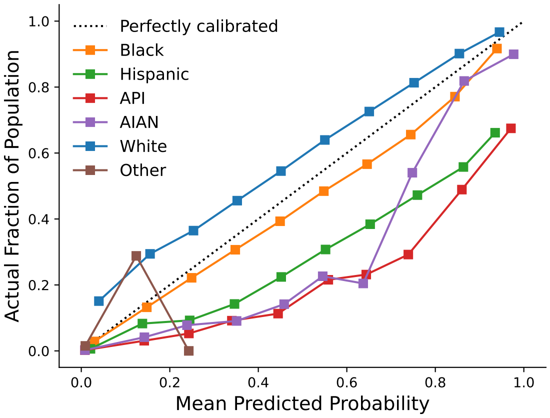


(c) Raking.

Figure S6: Replicating the calibration plots in Figure S4 with the data and predicted probabilities in Greengard and Gelman [2024].

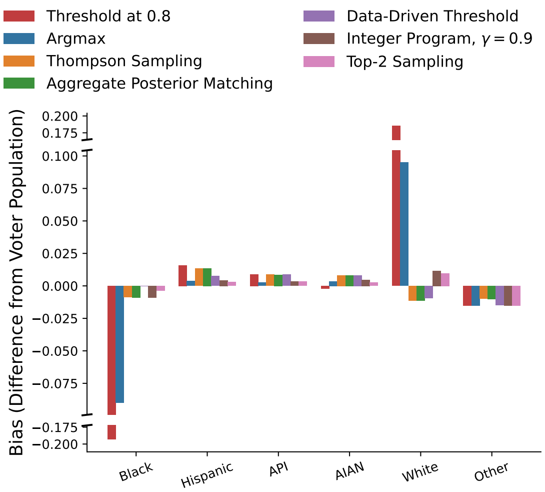


(a) Voterfile BISG.

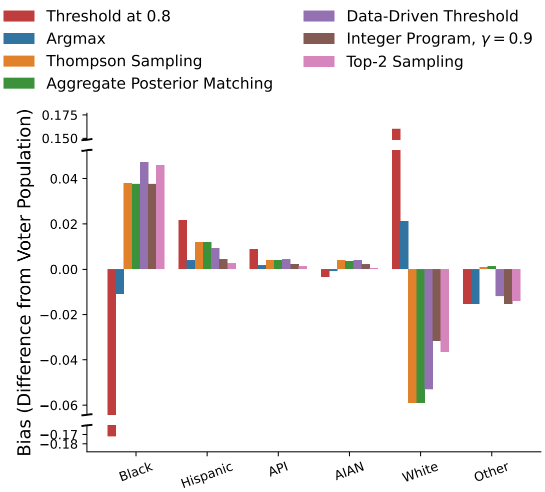


(b) Census BISG.

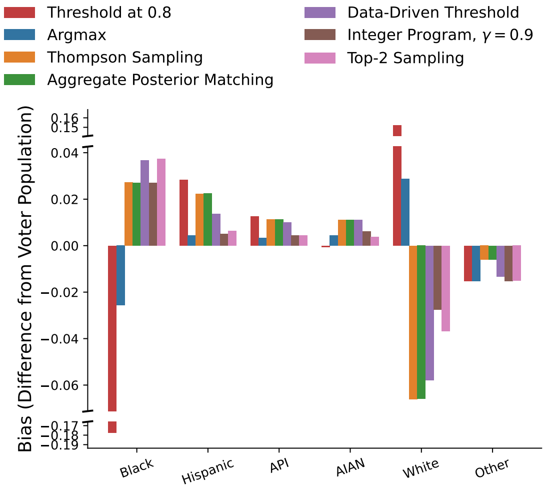


(c) Raking.

Figure S7: Replicating the bias plots in Figure S5 with the data and predicted probabilities in Greengard and Gelman [2024].

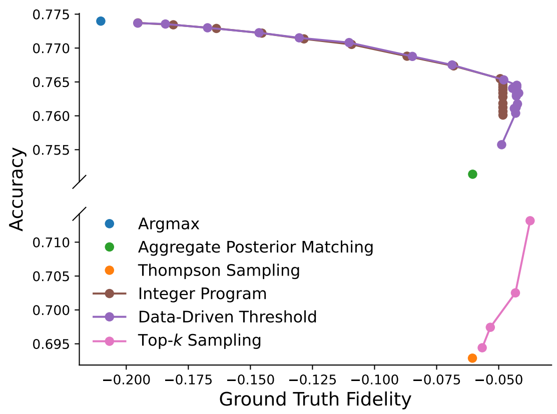


(a) Voterfile BISG.

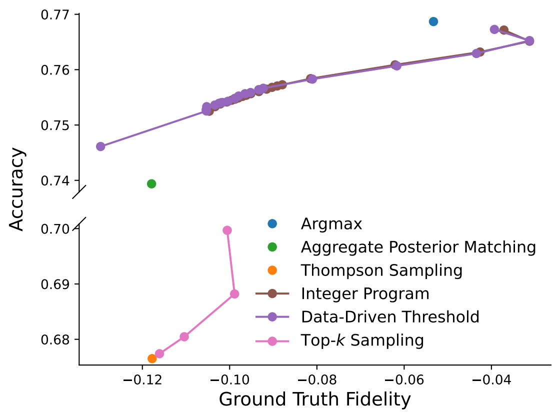


(b) Census BISG.

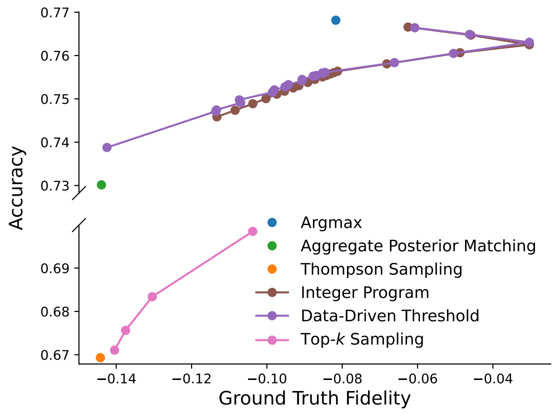


(c) Raking.

Figure S8: Replicating Figure 2c with the data and predicted probabilities in Greengard and Gelman [2024]. Note that in Figure S8c, the aggregate posterior is further from the ground truth distribution than the argmax solution, as the predictive model underrepresents the white majority (see Figure S6c), and so moving methods that increase fidelity to the Aggregate Posterior actually decrease fidelity to the Ground Truth.

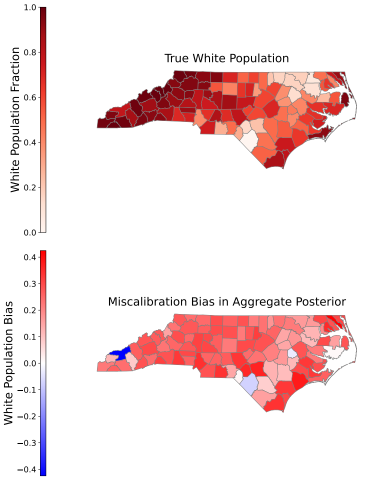


(a) White population and miscalibration bias in Voterfile BISG.

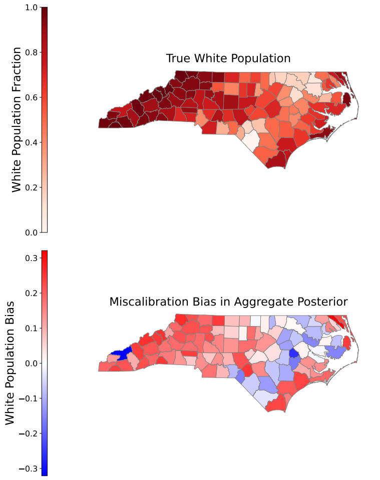


(b) White population and miscalibration bias in Census BISG.

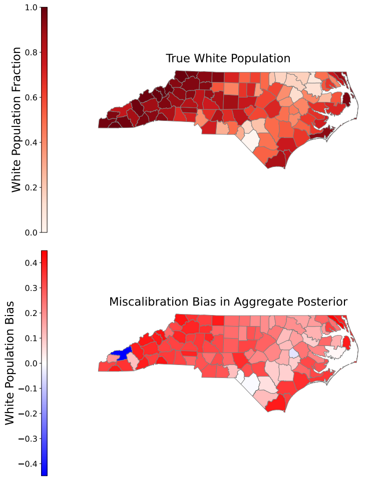


(c) White population and miscalibration bias in raking model.

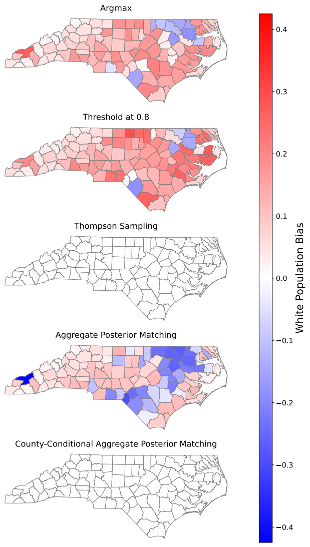


(d) Voterfile BISG.

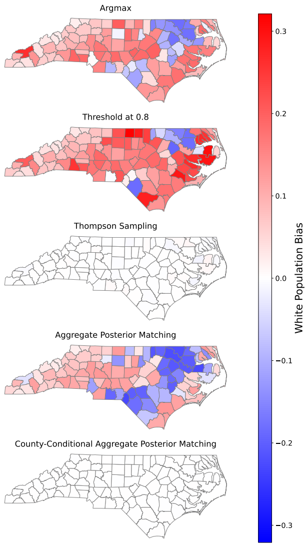


(e) Census BISG.

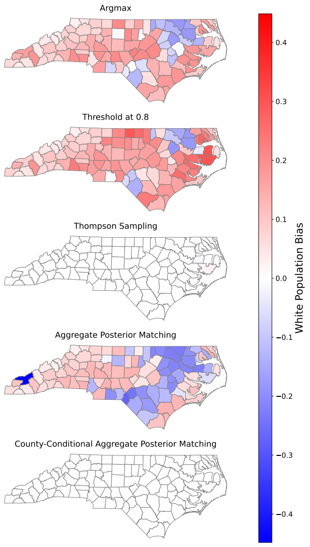


(f) Raking.

Figure S9: Replication of Figure 3 using the public data and predictions from Greengard and Gelman [2024].

| | Accuracy with Ground Truth | Fidelity to Ground Truth | Fidelity to Aggregate Posterior |
|---|---|---|---|
| Threshold at 0.8 (Among 53.4% Not Dropped) | **0.904** | -0.420 | -0.398 |
| Integer Program, $\gamma = 0.9$ | 0.765 | -0.048 | -0.046 |
| Argmax | 0.774 | -0.210 | -0.213 |
| Aggregate Posterior Matching | 0.751 | -0.061 | **-0.000** |
| Data-Driven Threshold Matching Heuristic | 0.756 | -0.049 | -0.021 |
| Thompson Sampling | 0.693 | -0.061 | -0.001 |
| Top-2 Sampling | 0.713 | -0.037 | -0.053 |
| Ground Truth Marginal Matching | 0.759 | **-0.000** | -0.060 |

(a) Voterfile BISG.

| | Accuracy with Ground Truth | Fidelity to Ground Truth | Fidelity to Aggregate Posterior |
|---|---|---|---|
| Threshold at 0.8 (Among 46.8% Not Dropped) | **0.915** | -0.381 | -0.466 |
| Integer Program, $\gamma = 0.9$ | 0.756 | -0.093 | -0.055 |
| Argmax | 0.769 | -0.053 | -0.160 |
| Aggregate Posterior Matching | 0.739 | -0.118 | **-0.000** |
| Data-Driven Threshold Matching Heuristic | 0.746 | -0.130 | -0.032 |
| Thompson Sampling | 0.677 | -0.118 | -0.000 |
| Top-2 Sampling | 0.700 | -0.101 | -0.061 |
| True Population Matching | 0.759 | **-0.000** | -0.118 |

(b) Census BISG.

| | Accuracy with Ground Truth | Fidelity to Ground Truth | Fidelity to Aggregate Posterior |
|---|---|---|---|
| Threshold at 0.8 (Among 45.9% Not Dropped) | **0.913** | -0.386 | -0.450 |
| Integer Program, $\gamma = 0.9$ | 0.755 | -0.085 | -0.077 |
| Argmax | 0.768 | -0.082 | -0.189 |
| Aggregate Posterior Matching | 0.730 | -0.144 | **-0.000** |
| Data-Driven Threshold Matching Heuristic | 0.739 | -0.142 | -0.035 |
| Thompson Sampling | 0.669 | -0.144 | -0.001 |
| Top-2 Sampling | 0.698 | -0.104 | -0.079 |
| Ground Truth Marginal Matching | 0.759 | **-0.000** | -0.144 |

(c) Raking.

Table S5: Replicating Table 1 with the data and predicted probabilities in Greengard and Gelman [2024].

## C.5 Replication on other states

We replicate our results (Figures S10 and S11 and table S6 corresponding to Figures 2a and 2c and table 1), with additional methods displayed, on similarly processed commercial voter file data from South Carolina ($N = 3{,}494{,}505$) and Florida ($N = 13{,}766{,}639$), which also report self-reported race. For further robustness, we also replicate on data from all three states joined together ($N = 23{,}635{,}780$), as well as all individuals across in the United States who have self-reported race/ethnicity data in the commercial file (i.e., including other states with self-reported race and former residents of NC/SC/FL that have moved elsewhere) ($N = 39{,}685{,}870$).

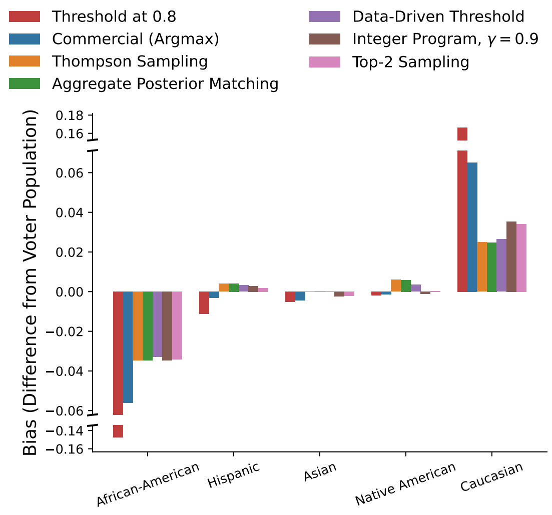


(a) South Carolina.

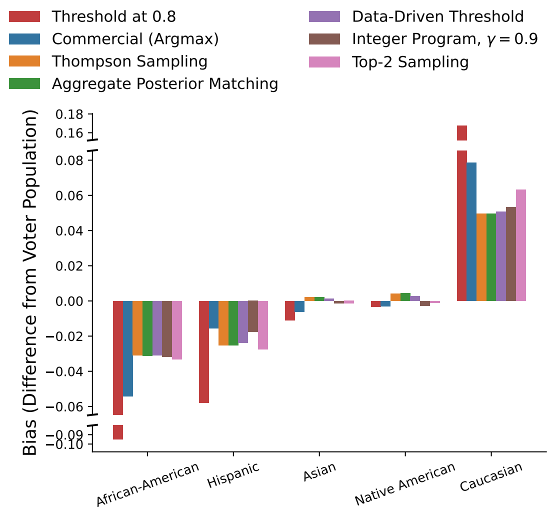


(b) Florida.

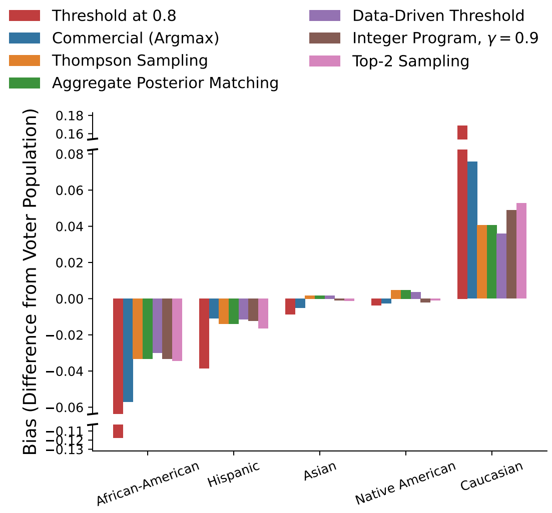


(c) North Carolina, South Carolina, and Florida combined.

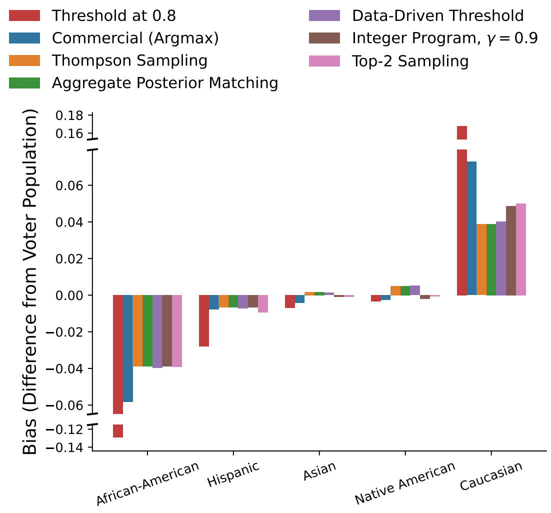


(d) All self-reported race data in the commercial voter file across the US.

Figure S10: Replication of Figure 2a with additional methods and data for other states from the commercial voter file.

| | Accuracy with Ground Truth | Fidelity to Ground Truth | Fidelity to Aggregate Posterior |
|---|---|---|---|
| Threshold at 0.8 (Among 66.8% Not Dropped) | **0.926** | -0.332 | -0.283 |
| Integer Program, $\gamma = 0.9$ | 0.845 | -0.076 | -0.021 |
| Commercial (Argmax) | 0.846 | -0.130 | -0.081 |
| Aggregate Posterior Matching | 0.838 | -0.069 | **-0.000** |
| Data-Driven Threshold Matching Heuristic | 0.840 | -0.066 | -0.007 |
| Thompson Sampling | 0.774 | -0.070 | -0.000 |
| Top-2 Sampling | 0.784 | -0.072 | -0.020 |
| Ground Truth Marginal Matching | 0.840 | **-0.000** | -0.069 |

(a) South Carolina.

| | Accuracy with Ground Truth | Fidelity to Ground Truth | Fidelity to Aggregate Posterior |
|---|---|---|---|
| Threshold at 0.8 (Among 70.8% Not Dropped) | **0.918** | -0.335 | -0.235 |
| Integer Program, $\gamma = 0.9$ | 0.848 | -0.107 | -0.022 |
| Commercial (Argmax) | 0.847 | -0.157 | -0.077 |
| Aggregate Posterior Matching | 0.841 | -0.112 | **-0.000** |
| Data-Driven Threshold Matching Heuristic | 0.842 | -0.109 | -0.005 |
| Thompson Sampling | 0.778 | -0.112 | -0.000 |
| Top-2 Sampling | 0.791 | -0.126 | -0.027 |
| Ground Truth Marginal Matching | 0.843 | **-0.000** | -0.112 |

(b) Florida.

| | Accuracy with Ground Truth | Fidelity to Ground Truth | Fidelity to Aggregate Posterior |
|---|---|---|---|
| Threshold at 0.8 (Among 69.4% Not Dropped) | **0.921** | -0.337 | -0.256 |
| Integer Program, $\gamma = 0.9$ | 0.847 | -0.098 | -0.020 |
| Commercial (Argmax) | 0.846 | -0.151 | -0.076 |
| Aggregate Posterior Matching | 0.842 | -0.094 | **-0.000** |
| Data-Driven Threshold Matching Heuristic | 0.843 | -0.083 | -0.012 |
| Thompson Sampling | 0.777 | -0.094 | -0.000 |
| Top-2 Sampling | 0.789 | -0.105 | -0.024 |
| Ground Truth Marginal Matching | 0.842 | **-0.000** | -0.094 |

(c) North Carolina, South Carolina, and Florida combined.

| | Accuracy with Ground Truth | Fidelity to Ground Truth | Fidelity to Aggregate Posterior |
|---|---|---|---|
| Threshold at 0.8 (Among 68.8% Not Dropped) | **0.924** | -0.336 | -0.258 |
| Integer Program, $\gamma = 0.9$ | 0.850 | -0.097 | -0.020 |
| Commercial (Argmax) | 0.848 | -0.146 | -0.068 |
| Aggregate Posterior Matching | 0.845 | -0.091 | **-0.000** |
| Data-Driven Threshold Matching Heuristic | 0.845 | -0.094 | -0.003 |
| Thompson Sampling | 0.778 | -0.091 | -0.000 |
| Top-2 Sampling | 0.789 | -0.100 | -0.023 |
| Ground Truth Marginal Matching | 0.845 | **-0.000** | -0.091 |

(d) All self-reported race data in the commercial voter file across the US.

Table S6: Replicating Table 1 with additional methods and data for other states from the commercial voter file.

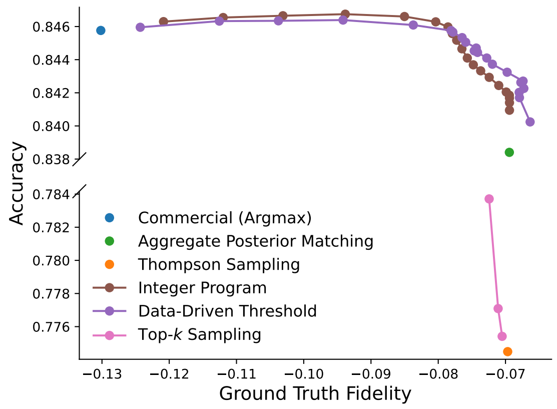


(a) South Carolina.

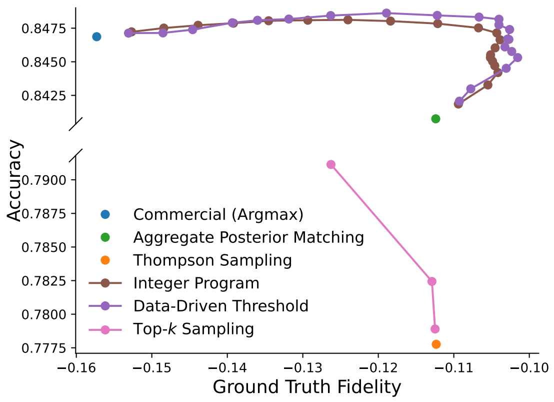


(b) Florida.

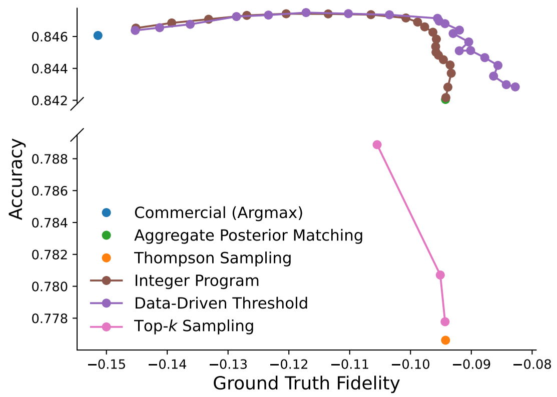


(c) North Carolina, South Carolina, and Florida combined.

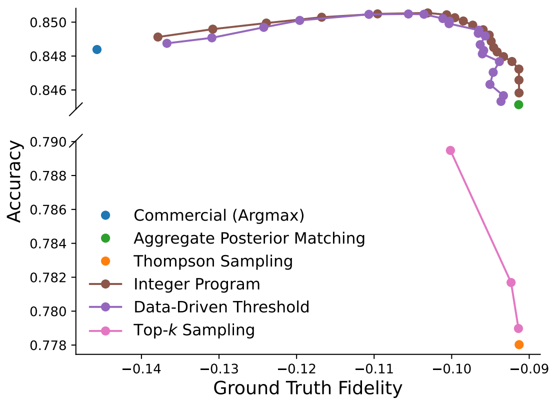


(d) All self-reported race data in the commercial voter file across the US.

Figure S11: Replication of Figure 2c with additional methods and data for other states from the commercial voter file.

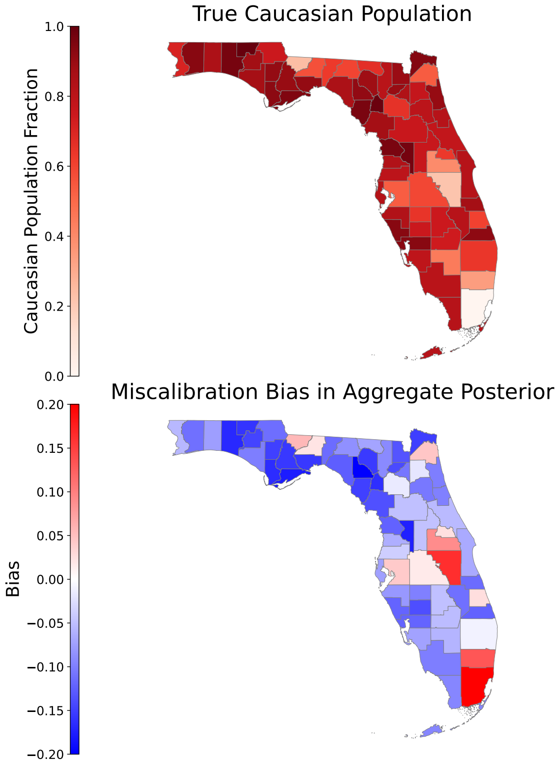


(a) White population and model miscalibration.

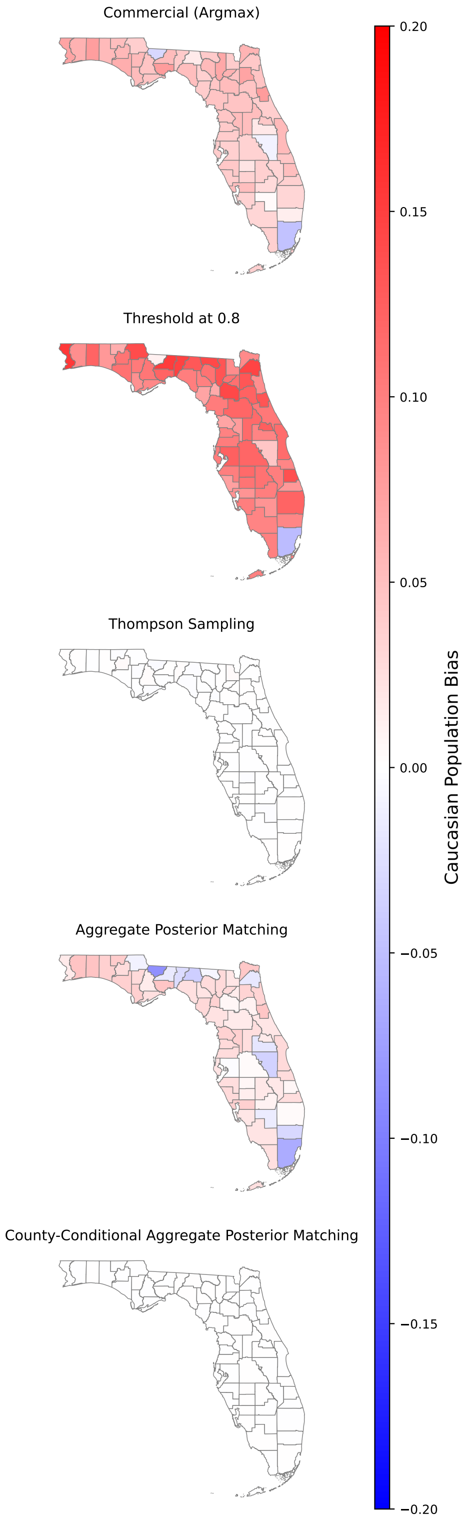


(b) Bias compared to per-county aggregate posterior.

Figure S12: Replication of Figure 3 in Florida with the commercial voter file.

## C.6 Voter File Results Without *Uncoded*

We now replicate our results with an alternative data processing choice: dropping the 205,683 entries that the commercial voter file labels as *Uncoded*, as opposed to replacing them with the argmax choice, leaving $N$ = 6,168,953 data points. Table S7 and Figure S13 replicate Table 1 and Figure 2, respectively. Results are qualitatively identical when comparing across decision rules. Note that the undercounting of voters of color is higher (percentage-wise) after dropping the *Uncoded* individuals, as these individuals tended to be (both in self-reported ground truth and imputed argmax) disproportionately voters of color. This follow the smae intuition seen in simpler threshold rules (e.g. probability at 0.8), where a preference for highly confident points leads to increased bias.

| | Accuracy with Ground Truth | Fidelity to Ground Truth | Fidelity to Aggregate Posterior |
|---|---|---|---|
| Threshold at 0.8 (30.6% Dropped) | **0.929** | -0.341 | -0.282 |
| Integer Program, $\gamma = 0.9$ | 0.853 | -0.093 | -0.034 |
| Commercial (Argmax) | 0.852 | -0.157 | -0.098 |
| Aggregate Posterior Matching | 0.842 | -0.076 | **-0.000** |
| Data-Driven Threshold Matching Heuristic | 0.844 | -0.076 | -0.007 |
| Thompson Sampling | 0.784 | -0.075 | -0.000 |
| Top-2 Sampling | 0.795 | -0.080 | -0.024 |
| Ground Truth Marginal Matching | 0.844 | **-0.000** | -0.075 |

Table S7: Replicating Table 1 with *Uncoded* labels removed as opposed to replaced with the argmax continuous scores.

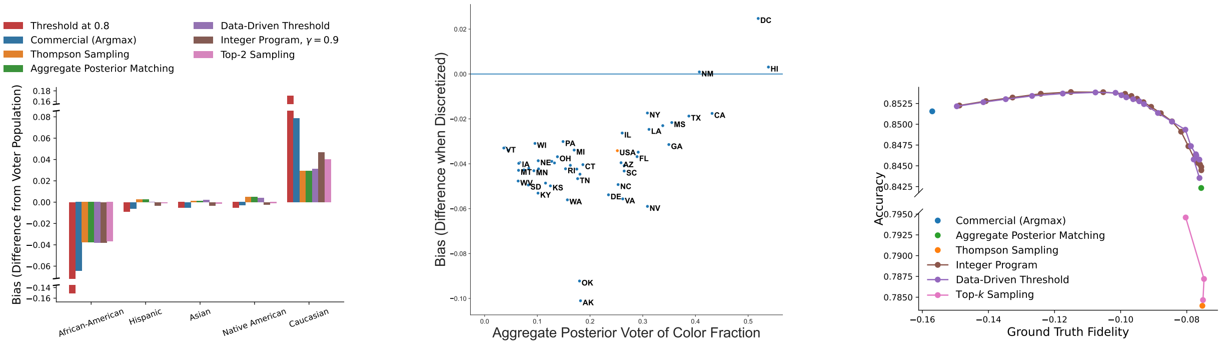


(a) Replication of Figure 2a

(b) Replication of Figure 2b

(c) Replication of Figure 2c

Figure S13: Replicating Figure 2 with the *Uncoded* category removed, $N$ = 6,168,953 registered voters. Note that in Figure S13b, many states across the board have lower counts for voters of color when data discretized when the *Uncoded* category is removed, as such data points tend to be less white than average.

## C.7 Additional Simplex Visualizations

In Figure S14, we provide additional visualizations for decision-making rules as a supplement to the figures in Figure 1.

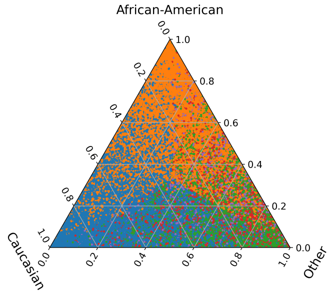


(a) Top-2 Sampling, randomized according to rescaled probabilities.

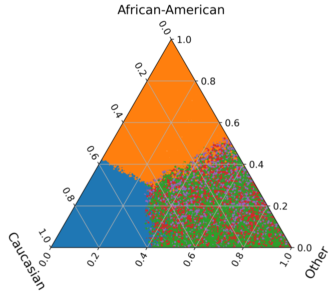


(b) Integer program, with chosen parameter $\gamma = 0.9$.

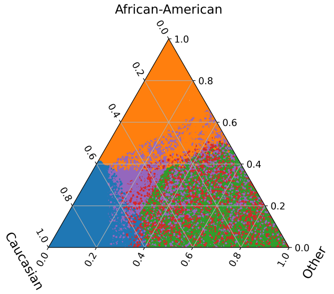


(c) Data-driven threshold heuristic, approximating matching from Figure 1d.

Figure S14: Extending Figure 1, classified labels onto a 3-dimensional probability simplex for other methods, with *Hispanic*, *Asian*, and *Native American* probabilities aggregated into *Other*.

# References


Himan Abdollahpouri, Zahra Nazari, Alex Gain, Clay Gibson, Maria Dimakopoulou, Jesse Anderton, Benjamin Carterette, Mounia Lalmas, and Tony Jebara. Calibrated Recommendations as a Minimum-Cost Flow Problem. In *Proceedings of the Sixteenth ACM International Conference on Web Search and Data Mining*, WSDM '23, page 571–579, New York, NY, USA, 2023. Association for Computing Machinery. ISBN 9781450394079. doi: 10.1145/3539597.3570402. URL `https://doi.org/10.1145/3539597.3570402`.

Dzifa Adjaye-Gbewonyo, Robert A Bednarczyk, Robert L Davis, and Saad B Omer. Using the Bayesian Improved Surname Geocoding Method (BISG) to Create a Working Classification of Race and Ethnicity in a Diverse Managed Care Population: A Validation Study. *Health Services Research*, 49(1):268–283, 2014.

McKane Andrus, Elena Spitzer, Jeffrey Brown, and Alice Xiang. What We Can't Measure, We Can't Understand: Challenges to Demographic Data Procurement in the Pursuit of Fairness. In *Proceedings of the 2021 ACM Conference on Fairness, Accountability, and Transparency*, pages 249–260, 2021.

Stephen Ansolabehere and Eitan Hersh. Gender, Race, Age, and Voting: A Research Note. In *APSA 2011 Annual Meeting Paper*, 2011.

Stephen Ansolabehere and Eitan Hersh. Validation: What Big Data Reveal about Survey Misreporting and the Real Electorate. *Political Analysis*, 20(4):437–459, 2012.

Lisa P Argyle and Michael Barber. Misclassification and Bias in Predictions of Individual Ethnicity from Administrative Records. *American Political Science Review*, 118(2):1058–1066, 2024.

Arthur P Baines and Marsha J Courchane. Fair Lending: Implications for the Indirect Auto Finance Market, Nov 2014. URL `https://www.crai.com/insights-events/publications/fair-lending-implications-indirect-auto-finance-market/`.

Michael Barber and Lisa P. Argyle. Replication Data for: Misclassification and Bias in Predictions of Individual Ethnicity from Administrative Records, 2023. URL `https://doi.org/10.7910/DVN/FEOKT6`.

Abeba Birhane, Vinay Uday Prabhu, and John Whaley. Auditing Saliency Cropping Algorithms. In *Proceedings of the IEEE/CVF Winter Conference on Applications of Computer Vision (WACV)*, pages 4051–4059, January 2022.

Emily Black, Rakshit Naidu, Rayid Ghani, Kit Rodolfa, Daniel Ho, and Hoda Heidari. Toward Operationalizing Pipeline-Aware ML Fairness: a Research Agenda for Developing Practical Guidelines and Tools. In *Proceedings of the 3rd ACM Conference on Equity and Access in Algorithms, Mechanisms, and Optimization*, pages 1–11, 2023.

William Cai, Johann Gaebler, Nikhil Garg, and Sharad Goel. Fair Allocation through Selective Information Acquisition. In *Proceedings of the AAAI/ACM Conference on AI, Ethics, and Society*, pages 22–28, 2020.

Simon Caton and Christian Haas. Fairness in Machine Learning: A Survey. *ACM Computing Surveys*, 2020.

Irene Chen, Fredrik D Johansson, and David Sontag. Why is My Classifier Discriminatory? *Advances in Neural Information Processing Systems*, 31, 2018.

Jiahao Chen, Nathan Kallus, Xiaojie Mao, Geoffry Svacha, and Madeleine Udell. Fairness Under Unawareness: Assessing Disparity when Protected Class is Unobserved. In *Proceedings of the Conference on Fairness, Accountability, and Transparency*, FAT* '19, page 339–348, New York, NY, USA, 2019. Association for Computing Machinery. ISBN 9781450361255. doi: 10.1145/3287560.3287594. URL `https://doi.org/10.1145/3287560.3287594`.

Matthew K Chin, Lan N Đoàn, Rienna G Russo, Timothy Roberts, Sonia Persaud, Emily Huang, Lauren Fu, Kiran Y Kui, Simona C Kwon, and Stella S Yi. Methods for Retrospectively Improving Race/Ethnicity Data Quality: A Scoping Review. *Epidemiologic Reviews*, page mxad002, 2023.

Rajashekar Chintalapati, Suriyan Laohaprapanon, and Gaurav Sood. Predicting Race and Ethnicity from the Sequence of Characters in a Name, 2023.

Consumer Financial Protection Bureau. Using Publicly Available Information to Proxy for Unidentified Race and Ethnicity: A Methodology and Assessment. *Washington, DC: CFPB, Summer*, 2014.

Sasha Costanza-Chock, Inioluwa Deborah Raji, and Joy Buolamwini. Who Audits the Auditors? Recommendations from a Field Scan of the Algorithmic Auditing Ecosystem. In *Proceedings of the 2022 ACM Conference on Fairness, Accountability, and Transparency*, pages 1571–1583, 2022.

Kevin DeLuca and John A. Curiel. Validating the Applicability of Bayesian Inference with Surname and Geocoding to Congressional Redistricting. *Political Analysis*, pages 1–7, 2022.

Rebecca Diamond, Tim McQuade, and Franklin Qian. The Effects of Rent Control Expansion on Tenants, Landlords, and Inequality: Evidence from San Francisco. *American Economic Review*, 109(9):3365–3394, 2019.

Marc N Elliott, Peter A Morrison, Allen Fremont, Daniel F McCaffrey, Philip Pantoja, and Nicole Lurie. Using the Census Bureau's Surname List to Improve Estimates of Race/Ethnicity and Associated Disparities. *Health Services and Outcomes Research Methodology*, 9:69–83, 2009.

Kevin Fiscella and Allen M Fremont. Use of Geocoding and Surname Analysis to Estimate Race and Ethnicity. *Health Services Research*, 41(4p1):1482–1500, 2006.

Bernard L Fraga. Candidates or Districts? Reevaluating the Role of Race in Voter Turnout. *American Journal of Political Science*, 60(1):97–122, 2016a.

Bernard L Fraga. Redistricting and the Causal Impact of Race on Voter Turnout. *The Journal of Politics*, 78(1):19–34, 2016b.

Nikhil Garg, Londa Schiebinger, Dan Jurafsky, and James Zou. Word Embeddings Quantify 100 Years of Gender and Ethnic Stereotypes. *Proceedings of the National Academy of Sciences*, 115(16):E3635–E3644, 2018.

Nikhil Garg, Hannah Li, and Faidra Monachou. Standardized Tests and Affirmative Action: The Role of Bias and Variance. In *Proceedings of the 2021 ACM Conference on Fairness, Accountability, and Transparency*, pages 261–261, 2021.

Yair Ghitza and Andrew Gelman. Voter Registration Databases and MRP: Toward the Use of Large-Scale Databases in Public Opinion Research. *Political Analysis*, 28(4):507–531, 2020.

Avijit Ghosh, Ritam Dutt, and Christo Wilson. When Fair Ranking Meets Uncertain Inference. In *Proceedings of the 44th International ACM SIGIR Conference on Research and Development in Information Retrieval*, pages 1033–1043, 2021.

Philip Greengard and Andrew Gelman. Replication Data for: BISG: When Inferring Race or Ethnicity, does it Matter that People Often Live Near Their Relatives?, 2023. URL `https://doi.org/10.7910/DVN/QIM4UF`.

Philip Greengard and Andrew Gelman. An Improved BISG for Inferring Race from Surname and Geolocation, 2024.

Jacob M Grumbach and Alexander Sahn. Race and Representation in Campaign Finance. *American Political Science Review*, 114(1):206–221, 2020.

Wenshuo Guo, Karl Krauth, Michael Jordan, and Nikhil Garg. The Stereotyping Problem in Collaboratively Filtered Recommender Systems. In *Equity and Access in Algorithms, Mechanisms, and Optimization*, pages 1–10, 2021.

Melissa Hall, Laurens van der Maaten, Laura Gustafson, Maxwell Jones, and Aaron Adcock. A Systematic Study of Bias Amplification. *arXiv preprint arXiv:2201.11706*, 2022.

Lisa Anne Hendricks, Kaylee Burns, Kate Saenko, Trevor Darrell, and Anna Rohrbach. Women Also Snowboard: Overcoming Bias in Captioning Models. In *Proceedings of the European conference on computer vision (ECCV)*, pages 771–787, 2018.

Kosuke Imai and Kabir Khanna. Improving Ecological Inference by Predicting Individual Ethnicity from Voter Registration Records. *Political Analysis*, 24(2):263–272, 2016.

Kosuke Imai, Santiago Olivella, and Evan TR Rosenman. Addressing Census Data Problems in Race Imputation via Fully Bayesian Improved Surname Geocoding and Name Supplements. *Science Advances*, 8 (49):eadc9824, 2022.

Abigail Z Jacobs and Hanna Wallach. Measurement and Fairness. In *Proceedings of the 2021 ACM Conference on Fairness, Accountability, and Transparency*, pages 375–385, 2021.

Meena Jagadeesan, Nikhil Garg, and Jacob Steinhardt. Supply-Side Equilibria in Recommender Systems. In *Thirty-seventh Conference on Neural Information Processing Systems*, 2023a. URL `https://openreview.net/forum?id=eqyhjLG5Nr`.

Meena Jagadeesan, Michael I Jordan, Jacob Steinhardt, and Nika Haghtalab. Improved Bayes Risk Can Yield Reduced Social Welfare under Competition. *arXiv preprint arXiv:2306.14670*, 2023b.

Shengyu Jia, Tao Meng, Jieyu Zhao, and Kai-Wei Chang. Mitigating Gender Bias Amplification in Distribution by Posterior Regularization. *arXiv preprint arXiv:2005.06251*, 2020.

Klas Leino, Matt Fredrikson, Emily Black, Shayak Sen, and Anupam Datta. Feature-Wise Bias Amplification. In *International Conference on Learning Representations (ICLR)*, 2019.

Zhi Liu and Nikhil Garg. Test-Optional Policies: Overcoming Strategic Behavior and Informational Gaps. *Equity and Access in Algorithms, Mechanisms, and Optimization*, pages 1–13, 2021.

Masoud Mansoury, Himan Abdollahpouri, Mykola Pechenizkiy, Bamshad Mobasher, and Robin Burke. Feedback Loop and Bias Amplification in Recommender Systems. In *Proceedings of the 29th ACM International Conference on Information & Knowledge Management*, pages 2145–2148, 2020.

Cory McCartan, Jacob Goldin, Daniel E Ho, and Kosuke Imai. Estimating Racial Disparities when Race is Not Observed. *arXiv preprint arXiv:2303.02580*, 2023.

Arvind Narayanan. Argmax Bias, 2021. URL https://twitter.com/random_walker/status/1399348241142104064. @random_walker.

Alejandro Noriega-Campero, Michiel A. Bakker, Bernardo Garcia-Bulle, and Alex 'Sandy' Pentland. Active Fairness in Algorithmic Decision Making. In *Proceedings of the 2019 AAAI/ACM Conference on AI, Ethics, and Society*, AIES '19, page 77–83, New York, NY, USA, 2019. Association for Computing Machinery. ISBN 9781450363242. doi: 10.1145/3306618.3314277. URL https://doi.org/10.1145/3306618.3314277.

Kenny Peng, Manish Raghavan, Emma Pierson, Jon Kleinberg, and Nikhil Garg. Reconciling the Accuracy-Diversity Trade-Off in Recommendations. In *The Web Conference*, 2024.

Emma Pierson, Camelia Simoiu, Jan Overgoor, Sam Corbett-Davies, Daniel Jenson, Amy Shoemaker, Vignesh Ramachandran, Phoebe Barghouty, Cheryl Phillips, Ravi Shroff, et al. A Large-Scale Analysis of Racial Disparities in Police Stops across the United States. *Nature Human Behaviour*, 4(7):736–745, 2020.

Sinan Seymen, Himan Abdollahpouri, and Edward C. Malthouse. A Constrained Optimization Approach for Calibrated Recommendations. In *Proceedings of the 15th ACM Conference on Recommender Systems*, RecSys '21, page 607–612, New York, NY, USA, 2021. Association for Computing Machinery. ISBN 9781450384582. doi: 10.1145/3460231.3478857. URL https://doi.org/10.1145/3460231.3478857.

Gabriel Stanovsky, Noah A. Smith, and Luke Zettlemoyer. Evaluating Gender Bias in Machine Translation. In Anna Korhonen, David Traum, and Lluís Màrquez, editors, *Proceedings of the 57th Annual Meeting of the Association for Computational Linguistics*, pages 1679–1684, Florence, Italy, July 2019. Association for Computational Linguistics. doi: 10.18653/v1/P19-1164. URL https://aclanthology.org/P19-1164.

Katelyn E Stauffer and Bernard L Fraga. Contextualizing the Gender Gap in Voter Turnout. *Politics, Groups, and Identities*, 10(2):334–341, 2022.

Harald Steck. Calibrated Recommendations. In *Proceedings of the 12th ACM Conference on Recommender Systems*, pages 154–162, 2018.

Rohan Taori and Tatsunori Hashimoto. Data Feedback Loops: Model-Driven Amplification of Dataset Biases. In Andreas Krause, Emma Brunskill, Kyunghyun Cho, Barbara Engelhardt, Sivan Sabato, and Jonathan Scarlett, editors, *Proceedings of the 40th International Conference on Machine Learning*, volume 202 of *Proceedings of Machine Learning Research*, pages 33883–33920. PMLR, 23–29 Jul 2023. URL https://proceedings.mlr.press/v202/taori23a.html.

Brian L Trippe, Buwei Huang, Erika A DeBenedictis, Brian Coventry, Nicholas Bhattacharya, Kevin K Yang, David Baker, and Lorin Crawford. Randomized Gates Eliminate Bias in Sort-Seq Assays. *Protein Science*, 31(9):e4401, 2022.

Ioan Voicu. Using First Name Information to Improve Race and Ethnicity Classification. *Statistics and Public Policy*, 5(1):1–13, 2018.

Angelina Wang and Olga Russakovsky. Directional Bias Amplification. In *International Conference on Machine Learning*, pages 10882–10893. PMLR, 2021.

Kyra Yee, Uthaipon Tantipongpipat, and Shubhanshu Mishra. Image Cropping on Twitter: Fairness Metrics, Their Limitations, and the Importance of Representation, Design, and Agency. *Proc. ACM Hum.-Comput. Interact.*, 5(CSCW2), oct 2021. doi: 10.1145/3479594. URL https://doi.org/10.1145/3479594.

Yan Zhang. Assessing Fair Lending Risks using Race/Ethnicity Proxies. *Management Science*, 64(1):178–197, 2018.

Dora Zhao, Jerone Andrews, and Alice Xiang. Men also do Laundry: Multi-Attribute Bias Amplification. In Andreas Krause, Emma Brunskill, Kyunghyun Cho, Barbara Engelhardt, Sivan Sabato, and Jonathan Scarlett, editors, *Proceedings of the 40th International Conference on Machine Learning*, volume 202 of *Proceedings of Machine Learning Research*, pages 42000–42017. PMLR, 23–29 Jul 2023. URL https://proceedings.mlr.press/v202/zhao23a.html.

Jieyu Zhao, Tianlu Wang, Mark Yatskar, Vicente Ordonez, and Kai-Wei Chang. Men Also like Shopping: Reducing Gender Bias Amplification Using Corpus-Level Constraints. In Martha Palmer, Rebecca Hwa, and Sebastian Riedel, editors, *Proceedings of the 2017 Conference on Empirical Methods in Natural Language Processing*, pages 2979–2989, Copenhagen, Denmark, September 2017. Association for Computational Linguistics. doi: 10.18653/v1/D17-1323. URL https://aclanthology.org/D17-1323.
